# Modes of action and diagnostic value of miRNAs in sepsis

**DOI:** 10.3389/fimmu.2022.951798

**Published:** 2022-08-05

**Authors:** Nikolaos Antonakos, Charly Gilbert, Charlotte Théroude, Irene T. Schrijver, Thierry Roger

**Affiliations:** Infectious Diseases Service, Department of Medicine, Lausanne University Hospital and University of Lausanne, Epalinges, Switzerland

**Keywords:** miRNA, sepsis, infection, innate immunity, biomarkers, critically ill

## Abstract

Sepsis is a clinical syndrome defined as a dysregulated host response to infection resulting in life-threatening organ dysfunction. Sepsis is a major public health concern associated with one in five deaths worldwide. Sepsis is characterized by unbalanced inflammation and profound and sustained immunosuppression, increasing patient susceptibility to secondary infections and mortality. microRNAs (miRNAs) play a central role in the control of many biological processes, and deregulation of their expression has been linked to the development of oncological, cardiovascular, neurodegenerative and metabolic diseases. In this review, we discuss the role of miRNAs in sepsis pathophysiology. Overall, miRNAs are seen as promising biomarkers, and it has been proposed to develop miRNA-based therapies for sepsis. Yet, the picture is not so straightforward because of the versatile and dynamic features of miRNAs. Clearly, more research is needed to clarify the expression and role of miRNAs in sepsis, and to promote the use of miRNAs for sepsis management.

## 1 Introduction

### 1.1 Innate immune sensing

Innate immune cells sense signals of microbial origin (microbial-associated molecular patterns or MAMPs, also known as pathogen-associated molecular patterns or PAMPs) or endogenous components released by injured or stressed cells (damage or danger-associated molecular patterns or DAMPs) through pattern-recognition receptors (PRRs). Lipopolysaccharide (LPS), peptidoglycan, flagellin, β-glucan, lipoproteins, glycoproteins, double-stranded and single-stranded RNA, and unmethylated CpG motif containing DNA from bacteria, mycoplasma, mycobacteria, fungi, parasites and viruses are MAMPs/PAMPs. The best described DAMPs are high mobility group box-1 (HMGB1), fibrinogen, fibronectin, nucleic acids, histones, heat shock proteins (HSPs), uric acid, ATP, cytochrome c, S100 molecules and serum amyloid A. The main families of PRRs comprise Toll-like receptors (TLRs), NOD-like receptors (NLRs), c-type lectin receptors, RIG-I-like receptors, cytosolic DNA sensors and scavenger receptors (1–4). The triggering of PRRs by MAMPs/DAMPs activates intracellular signal transduction pathways such as the nuclear factor-κB (NF-κB), interferon (IFN) response factor (IRF), mitogen-activated protein kinase (MAPK) and phosphoinositide 3-kinase/Akt/mammalian target of rapamycin (PI3K/Akt/mTOR) pathways regulating the expression of cytokines, acute phase proteins, and adhesion, co-stimulatory and major histocompatibility complex molecules as well as metabolism. A fine control of these pathways is essential to restore homeostasis following injury.

### 1.2 Sepsis

Sepsis-3 alliance redefined sepsis as “a life-threatening organ dysfunction caused by a dysregulated host response to infection” (5). Sepsis remains one of the leading causes of mortality worldwide. Recent estimations indicate that sepsis affects around 50 million people and is responsible of at least 11 million deaths annually worldwide (6). These numbers increased during the COVID-19 pandemic. Indeed, most patients dying from COVID-19 present respiratory failure (mostly acute respiratory distress syndrome, ARDS) and multi-organ failure, which are manifestations of sepsis (5). Despite progresses in basic, clinical and translational research, the pathophysiology of sepsis remains not fully understood. Sepsis-specific targeting strategies tested in clinical trials failed to show benefit for patients (7–19).

**Figure 1:**
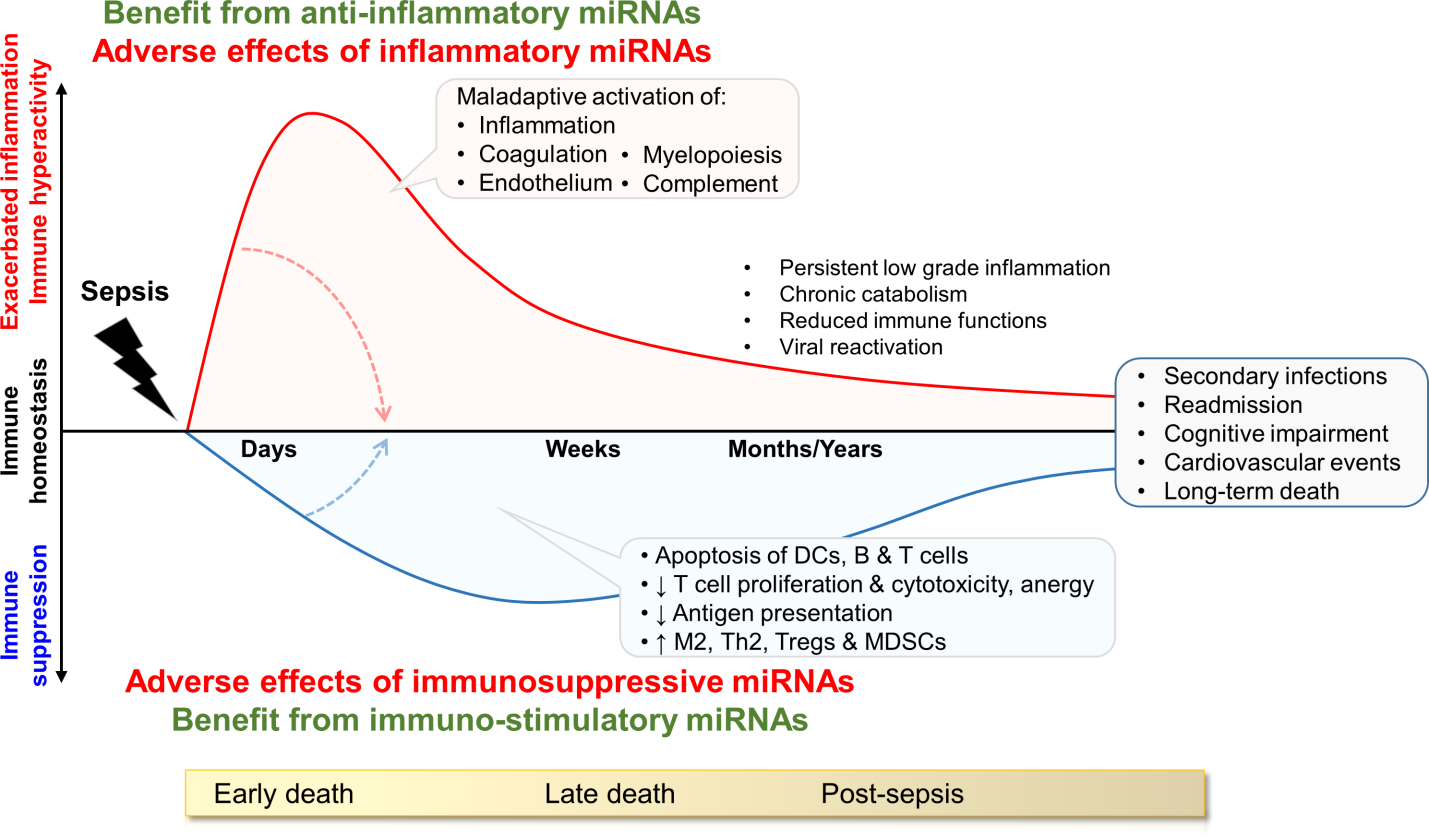
Model of immune status during sepsis and potential impact of miRNAs. The drawing shows the dysregulation of immune homeostasis over time, and lists pathophysiological consequences. The inflammatory and immunosuppressive responses are represented concurrently. Early deaths are mainly attributed to organ failure due to overwhelming inflammation. Late deaths are associated to immunosuppression causing increased susceptibility to (nosocomial) infections, viral reactivation and cardiovascular diseases. The influence of miRNAs may fluctuate over time. During the hyper-inflammatory phase of early sepsis, anti-inflammatory miRNAs can provide benefit to the host by dampening excessive immune reactions. In the immunosuppressive late phase of sepsis, inflammatory/immuno-stimulatory miRNAs can be beneficial by sustaining immune activity and protecting from nosocomial infections and reinfections. DCs: dendritic cells, MDSCs: myeloid derived suppressor cells; Th2: T helper 2, Tregs: regulatory T cells. M2 are pro-resolving/anti-inflammatory M2 macrophages.

Sepsis is characterized by an exacerbation of antimicrobial defense mechanisms responsible for collateral tissue injury, organ dysfunctions and early mortality involved in around 10% of all fatal cases (**Figure 1**). The hyper-inflammatory response is associated with a concurrent shift towards inflammation resolution and tissue repair involved in immuno-paralysis or immunosuppression. The suppressive phase is related to the depletion of dendritic cells (DCs), T cells and B cells through apoptosis, a reduced expression of proinflammatory cytokines, costimulatory and antigen-presenting molecules, and an increased expression of anti-inflammatory cytokines and inhibitory checkpoint molecules. Immunosuppression can persist for months to years (**Figure 1**). A subset of patients with prolonged stay in intensive care units (ICUs) suffer from persistent inflammation, immunosuppression and catabolism syndrome (PICS) (20). Dysregulated immune responses favor the development of secondary infections, viral reactivation and long-term immune disabilities accounting for late morbidity and mortality (7–13, 20). Delayed mortality associated with viral reactivation and nosocomial infections represent 20-40% and long-term mortality 50-70% of total fatal sepsis cases. Twenty percent of sepsis survivors develop secondary infections within 30 days, and nearly half of sepsis survivors are re-hospitalized within a year.

The identification of biomarkers and targets is one the most burning areas of research in the sepsis field. A biomarker is “any substance, structure, or process that can be measured in the body or its products and influence or predict the incidence of outcome or disease” (21). The identification of diagnostic, prognostic and theragnostic biomarkers to distinguish sepsis, identify patients who may benefit from host-targeted therapies, predict responsiveness and monitor the effectiveness of treatment holds great promise for improving patient management (10, 12, 22–29). In the last years, microRNAs (miRNAs) have been suggested to be potential biomarkers and targets for sepsis.

In this review we aim to shed light on the role of miRNAs involved in the pathogenesis of severe infections and sepsis. We will start by briefly summarizing the biogenesis, modes of action, circulation and delivery of miRNAs, which are described comprehensively elsewhere (30–35).

## 2 miRNAs

### 2.1 Identification

Non-coding RNAs (ncRNAs) comprise a growing list of RNA species, including miRNAs, small interfering RNAs, long non-coding RNAs (lncRNA), Piwi-interacting RNAs, small nuclear RNAs, small nucleolar RNAs, extracellular RNAs and small Cajal body-specific RNAs. ncRNAs regulate numerous biological and pathological processes such as cancer and autoimmune, cardiovascular and metabolic diseases.

In 1993, Lee et al. and Wightman et al. described a small RNA of 22 nucleotides, *lin-4*, with antisense complementarity to the heterochronic gene *lin-14* in *Caenorhabditis elegans* (36, 37). In 2000, the description of *let-7, a* small RNA conserved in diverse species and with silencing abilities, highlighted the critical role of this category of RNA molecules (38–40). The following year, the term microRNA was coined by Tuschl et al. (41). Along with other groups, they paved the way for the discovery of numerous miRNAs. About 38’600 miRNAs have been identified in 271 species (http://www.mirbase.org). Around 2’600 human mature miRNAs are encoded in the human genome, with half annotated in miRBase V22 (42). The expression atlas of miRNAs generated by the Functional Annotation of the Mammalian Genome (FANTOM5) consortium revealed that the five most expressed miRNAs represent around 50% of the miRNA pool in a given human cell type (43). About half of miRNAs are cell type-enriched, a quarter are broadly expressed, and a quarter are expressed at small levels regardless the cell type.

### 2.2 Biogenesis

**Figure 2:**
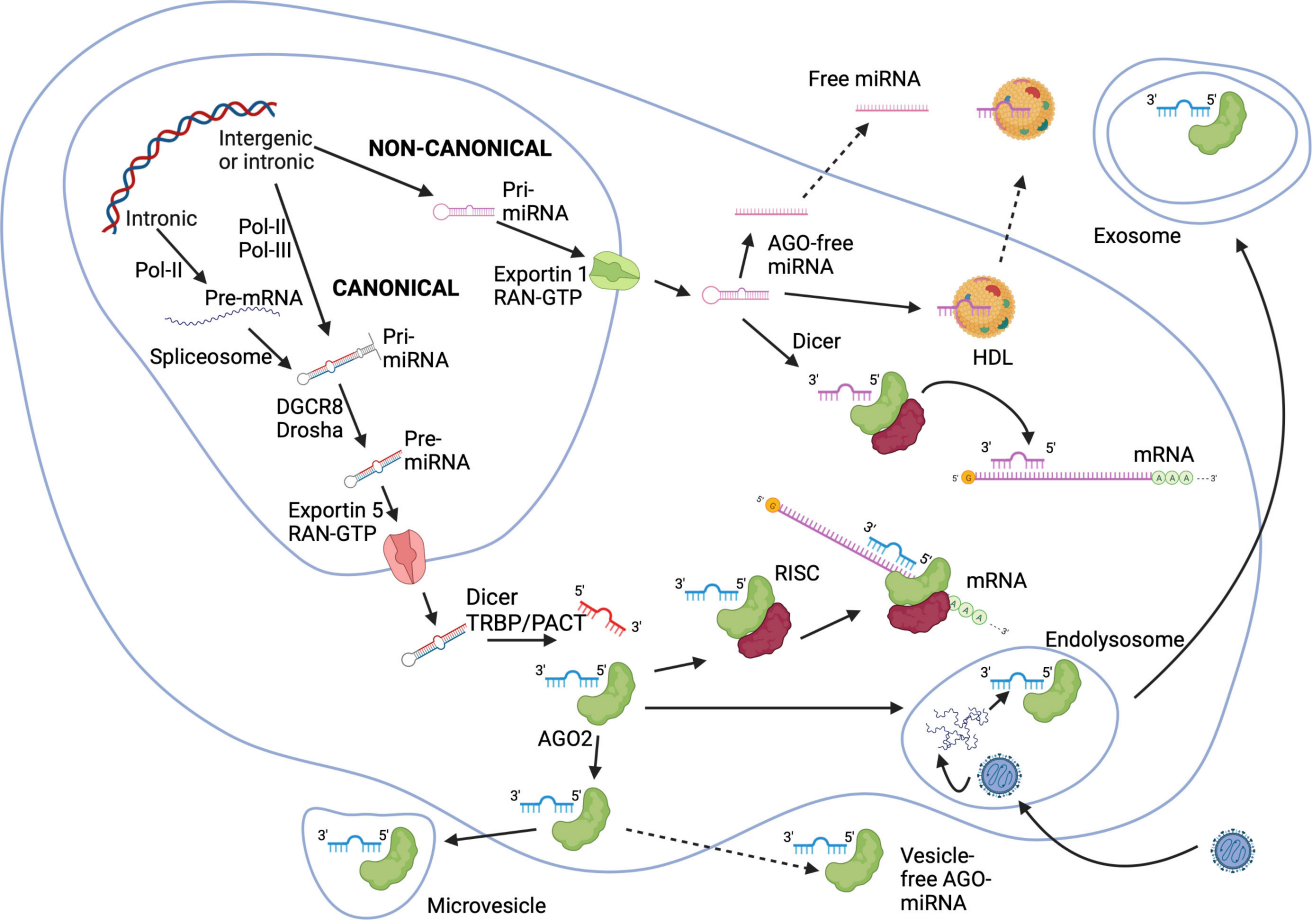
miRNA biogenesis *via* canonical and non-canonical pathways. In the canonical pathway, pri-miRNAs are turned into pre-miRNAs by the action of DGCR8 and Drosha within the nucleus. Intronic miRNAs can originate from host mRNA transcripts and processed into pre-miRNA by the spliceosome. Pre-miRNAs are exported into the cytoplasm through an exportin-5/RanGTP-dependent way, and are processed into mature miRNAs by Dicer with eventually RNA binding protein cofactors TRBP or PACT. In non-canonical pathways, shRNAs are cleaved by the DGCR8/Drosha complex and exported into the cytoplasm by exportin-1 before Dicer processing. Mature miRNAs bind to AGO proteins forming RISCs, which in turn silence or cleave mRNAs. Alternatively, miRNA-AGO complexes are exported out of the cell *via* vesicles (exosomes or microvesicles) or as vesicle-free complexes. miRNAs binding to HDLs are actively secreted. AGO-free miRNAs can be exported out of the cell as well. AGO, Argonaute; DGCR8, DiGeorge syndrome critical region gene 8; HDL, high density lipoproteins; miRNA, microRNA; mRNA, messenger RNA; PACT, protein kinase RNA activator; pre-miRNA, precursor-miRNA; Ran, Ras-related nuclear protein; RISC, RNA induced silencing complex; shRNA, small hairpin RNA; TRBP, transactivation response RNA binding protein. The Figure was created on BioRender.com.

miRNAs can be encoded in non-coding (intergenic miRNAs) and intronic regions of genes. miRNAs are generated through canonical and non-canonical pathways (32, 44, 45) (**Figure 2**). In the canonical pathway, a long primary transcript (pri-miRNA) of hundreds to thousands nucleotides is generated by RNA polymerase II (Pol-II) or Pol-III and cleaved through the action of the RNA-binding protein DiGeorge syndrome critical region gene 8 (DGCR8) and the nuclear RNase III enzyme Drosha into a precursor-miRNA (pre-miRNA) of approximately 70 nucleotides (46–49). Intronic pri-miRNAs are generated from host RNA transcripts (pre-mRNAs) by RNA splicing and excised into pre-miRNAs by spliceosomal components. Their expression relies on transcription factors and Pol-II (50). pre-miRNAs are exported into the cytoplasm in an exportin-5/RanGTP-dependent manner. Pre-miRNAs are converted into active miRNAs of approximatively 22 nucleotides by a complex composed of the cytoplasmic RNase III Dicer and cofactors including transactivation response (TAR) RNA binding protein (TRBP) and the protein kinase RNA activator (PACT) (49, 51, 52). Of note, miRNAs (-5p and -3p) can be generated from the 5’ and 3’ arms of a pre-miRNA precursor, and co-expression of miRNA-5p and -3p species have been repeatedly reported. Non-canonical miRNA biogenesis pathways use different combinations of proteins, and are grouped into DGCR8/Drosha-independent and Dicer-independent pathways (**Figure 2**). Small hairpin RNA (shRNA) are cleaved by the DGCR8/Drosha complex and exported into the cytoplasm as in the canonical pathway, while pre-miRNA can be exported into the cytoplasm through exportin-1. A more detailed description of miRNA biogenesis pathways is beyond the scope of this review, but available in excellent reviews (32–35).

### 2.3 Modes of action

miRNAs interact with the 3′-untranslated region (3’-UTR) of mRNAs to induce mRNA degradation and translational repression. Additionally, miRNAs can interact with gene promoter, 5′-untranslated region (5’-UTR) and coding sequence, and can activate transcription in a phenomenon known as RNA activation (53). Finally, miRNA can interact with proteins to modify their activity.

Crosslinking and immunoprecipitation analyses revealed that most miRNA binding events have little functional consequences (54). miRNAs do not possess catalytic functions, but form effector ribonucleoprotein complexes known as RNA induced silencing complexes (RISCs) (55). Mature miRNA molecules bind with proteins of the Argonaute (AGO) family in an ATP-dependent manner. Four AGO proteins (AGO1-4) playing a key role in the formation of RISCs are expressed in humans (56). RISCs bind to target mRNA molecules based on complementarity of miRNA (**Figure 2**). The result can be translational inhibition by interfering with the eukaryotic initiation factor 4F (eIF4F) followed by the decay of the target mRNA. Moreover, AGO2 initiates mRNA deadenylation by poly(A)-deadenylases, uncapping and 5′−3′ degradation by an exoribonuclease (32, 55, 57). While full complementarity with the target mRNA triggers AGO2 and mRNA degradation, partial complementarity results in transient binding to RISC. It induces the unloading of miRNA from AGO2 (57, 58). AGO-free miRNA molecules and endogenous miRNA-mRNA duplexes have been studied during the past years (59, 60). Mature miRNAs may adopt secondary structures like hairpin and homoduplex that may increase their half-life, affinity and specificity for targets (61).

Free miRNAs interact with proteins, but the prevalence and outcome of such interactions are poorly described. For instance, miR-130b-3p binds to extracellular cold-inducible RNA binding protein (eCIRP) (62). miR-130b-3p and eCIRP are increased in the blood of septic mice and sepsis patient. eCIRP acts as a DAMP sensed through TLR4, promoting the release of inflammatory mediators. Upon binding to eCIRP, miR-130b-3p inhibits eCIRP/TLR4 interaction and cytokine release by immune cells. Injection of a miR-130b-3p mimic reduces cecal ligation and puncture (CLP)-induced inflammation and acute lung injury (ALI) in mice (62). Moreover, miR-130b-3p has been shown to inhibit M1 macrophage polarization (63). A single miRNA can thus interfere with immune responses through multiple ways.

### 2.4 Circulation and delivery

miRNA secretion and release are intrinsic to cell response to hypoxia, starvation, heat, triggering of PRRs and cytokine/growth factor receptors, and other environmental factors (32, 59, 64, 65). miRNAs are present in biological fluids like blood, plasma, serum, urine, tears, saliva, semen, cerebrospinal fluid, bronchial and peritoneal fluids, and breast milk (32, 59, 64). An important feature of extracellular miRNAs is their stability and resistance to RNaseA-mediated degradation (64, 66). miRNAs in fluids exert paracrine or endocrine effects as signal transducers of intracellular communication (32, 65).

miRNAs are released passively accompanying apoptotic bodies or cell debris (from one to few μm) or secreted actively (59, 65). Secretion occurs through microvesicles of 100 to 1000 nm usually containing a RISC or a miRNA-AGO complex, and through exosomes (59). MAMPs or DAMPs trigger the release of exosomes containing miRNAs as well as DAMPs such as HMGB1, HSPs and histones, and cytokines, interleukins (ILs), chemokines and IFNγ. Early endosomes gradually turn to multivesicular bodies that integrate miRNAs and a RISC or similar complexes through mechanisms regulated by ceramide synthesis and neutral sphingomyelinase 2 (65, 67). Multivesicular bodies merge with lysosomes inducing the degradation of trapped material, or fuse with the cell membrane expelling exosomes containing miRNAs (59, 68). Exosomes can carry oncogenic miRNAs promoting tumor invasiveness (69), or anti-oncogenic and anti-angiogenic miRNAs inhibiting the growth of malignant cells (70). Similarly, exosomes can carry miRNAs that enhance or decrease cellular responses to MAMPs as reported for miR-155 and miR-146a in LPS-stimulated DCs (71). High-density lipoproteins (HDL) act as alternative carriers of miRNAs in the blood (**Figure 2**). This is an active and energy dependent procedure to differentiate from the passive release of miRNAs upon cell death (65).

The mechanisms of uptake of miRNAs by recipient cells is not fully deciphered (65). The uptake of microvesicles and exosomes occurs by endocytosis, phagocytosis or fusion with the plasma membrane. Endocytosis of microvesicles requires a docking step mediated by specific or non-specific molecules (72, 73). Because of their small size, microvesicles are also taken-up by micropinocytosis, which does not require a docking step (74). Exosomes and smaller extracellular vesicles are engulfed by phagocytosis mediated by TLRs and complement receptors. Exosomes released during sepsis impact on organs including lungs, kidneys, liver, heart and brain (75).

## 3 miRNAs in sepsis

Sepsis shows features of early immune hyper-activation and late immunosuppression. Accordingly, we may suggest that miRNAs having anti-inflammatory activities may be beneficial during early sepsis but detrimental during late sepsis. On the contrary, miRNAs having proinflammatory activities may be detrimental during early sepsis but beneficial during late sepsis (**Figure 1**).

Given that infection and stress modulate the expression of miRNAs, it is not surprising that miRNAs have been the focus of much interest. The stability, simple structure and expression of miRNAs in blood and other biological fluids represent an opportunity to stem new sepsis biomarkers (76). We will focus on promising miRNAs in sepsis. We will summarize observations about the modulation and the role of miRNAs *in vitro* and *in vivo* in models of sepsis (**Table 1**), and miRNAs as potential biomarkers in human sepsis (**Table 2**).

**Table 1:** Selection of miRNAs related to sepsis.

miRNA	Expression/model	Target and effect	Observation/impact of miRNA	Reference
miR-15a/16	Increased in BMDMs exposed to LPS	↓ PU.1 & TLR4	Decreased phagocytic and bactericidal activities of BMDMs Decreased inflammatory response Increased survival of miR-15a/16 knockout mice with sepsis (CLP and *E. coli* peritonitis)	(77)
miR-15a/16	RAW 264.7 mouse macrophages exposed to LPS	↓ TLR4 & IRAK1		(78)
miR-15a-5p	Increased in RAW 264.7 mouse macrophages exposed to LPS	↓ TNIP2 ↑ NF-κB pathway	Increased expression of IL-1β, IL-6 and TNF miR-15a-5p inhibitor reduced cytokines and inflammation in mice challenged with LPS	(79)
miR-15-5p, miR378a-3p	Expressed in platelet-derived exosomes from sepsis patients	↓ PDK1	Modulate Akt/mTOR-related autophagy pathway and induced NETs formation involved in organ injury	(80)
miR-16	Increased in H69 human biliary epithelial cells and U-937 human monocytic cells exposed to LPS	↑ NF-κB pathway by suppressing SMRT	Increased expression of IL-1β, IL-6 and TNF	(81)
miR-17	Decreased in RAW 264.7 macrophages exposed to LPS	↓ BRD4	Inhibition of BRD4 /EZH2/TRAIL pathway Decreased inflammatory response of RAW 264.7 macrophages	(82)
miR-19a	Increased in B cells of sepsis patients	CD22 ↑ 2 days after LPS stimulation CD22 ↓ 4 days after LPS stimulation	Positive feedback loop of B cell response	(83)
miR19b	HEK293T and HeLa cells, MEFs, human synovial fibroblasts	↑ NF-κB pathway by suppressing A20/Tnfaip3, Rnf11, Fbxl11/Kdm2a and Zbtb16	Increased production of IL-6 and IL-8	(84)
miR-21	Increased in serum of pediatric sepsis patients	↑ NF-κB pathway & NLRP3 inflammasome	Induction of pyroptosis in mouse macrophages, human THP-1 monocytic cells & primary PBMCs via activation of the NLRP3 inflammasome	(85)
miR-21	Increased in bone marrow of sepsis mice (CLP)	↑ NFI-A protein	Increased number of MDSCs by arresting myeloid progenitor differentiation and maturation miR-21 antagomiR improves late-sepsis survival	(86)
miR-21	High levels in MDSCs from sepsis mice (CLP)	–	miR-21 up-regulated by STAT3 and C/EBPβ in MDSCs Involved in the expansion of MDSCs	(87)
miR-23a	Decreased in bone marrow mononuclear cells of sepsis mice	↓ lncRNA MALAT1 & MCEMP1	Decreased proliferation of monocytes Increased apoptosis of monocytes	(88)
miR-23a	Decreased in RAW 264.7 mouse macrophages exposed to LPS	↓ ATG12	Decreased autophagy, increased production of IL-6 and TNF	(89)
miR-23a-3p	Decreased in RAW 264.7 mouse macrophages exposed to LPS	↓ PLK1	Increased STAT1/STAT3 activation, TNF, IL-1β, IL-6 production and M1 polarization by macrophages Promotes ALI in mice challenged intratracheally with LPS	(90)
miR-26a	Decreased in serum and mononuclear of sepsis neonates	↓ IL-6		(91)
miR-26b	Decreased in MEG-01 human megakaryocyte cells exposed to LPS or TNF	–	Associated with ↑ expression of SLEP Downregulation of Dicer1 reduced miR-26b and increased SELP	(92)
miR-27a	Down-regulation by TUG1 (possible “sponge” action) in human cardiomyocyte cell line AC16	↓ TNF	LPS up-regulates miR-27a and down-regulates TUG1 TUG1 overexpression inhibits TNF and apoptosis of AC16 cells	(93)
miR-27a	Increased in lung tissues of sepsis mice	↑ TNF, IL-6	miR-27a neutralization decreases pulmonary inflammation and increases survival of sepsis mice	(94)
miR-27b	Increased in MSCs-derived exosomes of sepsis mice	↓ JMJD3 & JMJD3/NF-κB/p65 axis	Inhibition of pro-inflammatory response of BMDMs after LPS stimulation and in CLP-induced sepsis model	(95)
miR-30a	Increased in liver cells of sepsis rats	↓ SOCS-1	Increased apoptosis of liver cells via JAK/STAT pathway	(96)
miR-30e	Decreased in liver cells and tissues of sepsis rats	↓ FOSL2	Decreased apoptosis of liver cells via inhibition of JAK/STAT pathway	(97)
miR-34a	Increased in lung tissues of sepsis mice (CLP)	↓ SIRT1 & ATG4B	Increased oxidative stress, inflammatory response and sepsis-induced ALI	(98)
miR-34a	Increased in lung macrophages of suckling rats after LPS stimulation	↑ iNOS, phospho-STAT3/STAT3	Increased sepsis-induced ALI Inhibition of miR-34a decreases sepsis-induced ALI	(99)
miR-92a-3p	Increased in the BALF of the sepsis rats	↓ PTEN	Increased activation of alveolar macrophages Increased ALI via PI3K/AKT pathway	(100)
miR-98	Decreased in myocardial tissues of sepsis mice (CLP)	↓ HMGA2, TNF, IL-6	Decreased sepsis-induced cardiac dysfunction, liver and lung injury	(101)
miR-103a-3p	Decreased in sepsis patients	↓ HMGB1	Decreased HMGB1 expression, systemic inflammation and multi organ failure, and increased survival in mice with endotoxemia	(102)
miR-122	Huh7 hepatocellular carcinoma cell line	↓ SOCS1	Increased expression of IFNα & IFNβ	(103)
miR-122	Huh7 cells	↓ SOCS3	Increased expression of IFNα & IFNβ Decreased HBV replication	(104)
miR-122	HepG2, Huh7 and Huh7.5.1 hepatocellular carcinoma cell lines	↓ FGFR1, IGF1R, MERTK ↑ IFN signaling pathway	Decreased STAT3 Tyr705 phosphorylation, increased IRF1 signaling and IFNs expression in response to HCV and poly (I:C)	(105)
miR-122	Huh-7 and HepG2 cells	↓ HO-1	Inhibit HBV expression (HBsAg and HBeAg expression)	(106)
miR-122	Increased in HepG2 and Huh7 cells	↓ TLR4	Decreased the proliferation and the production of TNF and IL-6 by HepG2 and Huh7 cells	(107)
miR-122-5p	Increased by LPS in the heart of rats and in H9c2 rat cardiomyocytes	–	Inhibition of miR-122-5p reduced myocardial injury through inhibiting inflammation, oxidative stress and apoptosis in endotoxemic rats	(108)
miR-124	Decreased in organs of mice with LPS-induced acute lung injury (ALI)	↓ MAPK14 (p38-α)	Overexpression decreases IL-1β, IL-6, IL-10 and TNF in blood, and MAPK signaling and lung cell apoptosis and lung injury in ALI mice	(109)
miR-125b	Decreased in PBMCs exposed to LPS, and in PBMCs and serum of sepsis patients	↓ STAT3	Inhibition in peripheral blood monocytes increases STAT3 phosphorylation and the expression of PCT and NO Possibly acts downstream the *NED25* gene intergenic lncRNA	(110)
miR-125b	Decreased in PBMCs exposed to LPS, and in PBMCs of sepsis patients	↓ STAT3	Decreased PCT	(111)
miR-125-5p	Decreased in mice with CLP and ALI	↓ TOP2A	Endothelial cell−derived exosomal miRNA−125b−5p ↑ VEGF, protected from sepsis−induced ALI	(112)
miR-126-3p in platelet microparticles	Increased in primary macrophages exposed to miR-126-3p-containing platelet microparticles		Decrease of 367 RNAs & reduced expression of CCL4, CSF1 and TNF, increase phagocytic capacity by macrophages	(113)
miR-126-5p	Increased in hepatic cells of mice with sepsis induced AHI	↓ BCL2L2	Overexpression inhibits anti-apoptotic function of BCL2L2 in AHI LncRNA-CRNDE binds miR-126-5p and restores BCL2L2 function	(114)
miR-128	Increased in kidneys of mice with sepsis induced AKI	↓ NRP1	NRP1 downregulates TNF, IL-6, and IL-1β NRP1 inhibition by miR-128 increases inflammatory response	(115)
miR-128-3p	Decreased in HK2 cells exposed to LPS and serum of sepsis patients	↓ TGFBR2	Decreased TGFBR2 mediated apoptosis	(116)
miR-129	Decreased in lungs of mice with LPS-induced ALI	↓ TAK1	Inhibits TAK1/NF-κB pathway Decreased apoptosis and inflammation in sepsis-induced ALI	(117)
miR-129-5p	Decreased in kidneys of mice with LPS-induced AKI	↓ HMGB1	Inhibits HMGB1/TLRs/NF-κB pathway Decreased apoptosis of kidney podocytes	(118)
miR-129-5p	Overexpression (use of agonists) in sepsis mice (CLP)	↓ HMGB1	Decreased HMGB1, apoptosis and inflammation in sepsis-induced ALI	(119)
miR-130a	Decreased in sepsis patients with thrombocytopenia	↓ IL-18	–	(120)
miR-130b-3p	Increased in serum of sepsis mice (CLP) and in sepsis patients	Binds to and inhibit CIRP	Decreased CIRP-induced cytokine production by macrophages Delivery of miR-130b-3p in mice reduces CLP-induced inflammation and acute lung injury	(62)
miR-130b-3p	Increased in RAW 264.7 mouse macrophages exposed to IFNγ+LPS	↓ IRF1	Inhibits M1 macrophage polarization and production of CCL5, CXCL-10, iNOS & TNF Overexpression decreases lung inflammation in LPS‐treated mice	(63)
miR-132	Increased in alveolar macrophages of sepsis rats	↓ AChE	Decreased ACh-mediated cholinergic anti-inflammatory reaction Inhibits NF-κB and STAT3	(121)
miR-133a	Decreased in TCMK-1 mouse kidney cell line exposed to LPS	↓ BNIP3L	Inhibits NF-κB pathway, apoptosis and TNF and IL-6 expression	(122)
miR-133a	Increased in the blood of sepsis mice (CLP) and sepsis patients	↓ SIRT1	Inhibition of miR-133a decreased CLP-induced inflammation and lung, liver and kidney injuries	(123)
miR-135a	Increased in serum of sepsis patients	↑ p38 MAPK	Activation of p38 MAPK and NF-κB pathways Aggravation of sepsis-induced inflammation and myocardial dysfunction	(124)
miR-139-5p	Decreased in lung tissues of sepsis mice (CLP)	↓ MyD88	Decreased inflammation, oxidative stress and ALI	(125)
miR-141	Decreased in serum of pediatric sepsis patients and in monocytes exposed to LPS	↓ TLR4	Decreased inflammatory response in neonatal sepsis	(126)
miR-142	Decreased in blood of sepsis patients and in macrophages of sepsis mice	↓ PD-L1	Decreased inflammation mediated by PD-L1	(127)
miR-143	Increased in the blood of healthy volunteers infused with LPS	–	Associated with strong reduction of BCL2 and silencing of inflammation related targets	(128)
miR-143 miR-150	Increased in mouse macrophages exposed to mycobacterial cell wall glycolipid and muramyl dipeptide	↓ TAK1 (miR-143) ↓ RIP2 (miR-150)	Negatively regulate the NOD2 pathway Suppress MDP-induced PI3K-PKC-MAPK-βcatenin-mediated expression of COX-2, SOCS3 and MMP-9	(129)
miR-143	Decreased in nasal mucosal tissues from patients with allergic rhinitis	↓ IL-13αR1	Decreased expression of GM-CSF, eotaxin and mucin 5AC of cells exposed to IL-13	(130)
miR-143	Decreased in HUVECs exposed to IL-1β	↓ ADAR1	Promotes the activation of HUVECs by IL-1β	(131)
miR-143	Increased in BEAS-2B human bronchial epithelium cells exposed to AngII and LPS	↓ ACE2	miR-143-3p inhibitor increased ACE2 and decreased IL-1β, IL-6 and TNF and apoptosis in cells exposed to AngII and LPS	(132)
miR-143	Decreased in lung tissues of mice with mycoplasmal pneumonia	↓ MyD88	miR-143 increased IL-10 and decreased IL-2, TNF and alveolar epithelial cell apoptosis through Bax and Bcl-2	(133)
miR-143	Human umbilical cord MSCs exposed to poly(I:C)	↓ TAK1 and COX-2	Infusion of TLR3-activated MSCs improved survival of sepsis mice (CLP); the co-infusion of miR-143 reduced survival benefit	(134)
miR-145	Decreased in blood samples of sepsis patients and in lung tissues of sepsis mice	↓ TGFBR2	Decreased LPS-induced inflammation and sepsis-induced ALI	(135)
miR-145	Decreased in HUVECs exposed to LPS	↓ TGFBR2	Decreased TGFBR2/SMAD2/DNMT1 pathway Decreased LPS-induced injury	(136)
miR-146	Decreased in EA.hy926 human vascular endothelial cells exposed to LPS	↓ NF-κB pathway	Decreased LPS-induced expression of inflammatory cytokines	(137)
miR-146a	Increased uptake of miR-146a-expressing plasmid by splenic macrophages of sepsis mice	↓ IRAK-1, TRAF6	Decreased sepsis-induced inflammation and organ failure Splenectomy abolishes these effects	(138)
miR-146a	Increased in peritoneal macrophages of sepsis mice after GSKJ4 treatment	–	Decreased expression of pro-inflammatory cytokines by JMJD3 inhibition after GSKJ4 treatment	(139)
miR-146a	Increased in heart-derived H9c2 cardiomyocytes exposed to LPS	↑ ErbB4 ↓ IRAK1, TRAF6, caspase-3	Decreased sepsis-induced inflammation and myocardial dysfunction	(140)
miR-146a	Increased in mouse peritoneal macrophages exposed to LPS	↓ Notch-1	Decreased NF-κB signaling Decreased sepsis-induced organ failure	(141)
miR-146a	Decreased in T cells of sepsis patients	↓ PRKCϵ	Decreased STAT4 activation via PRKCϵ downregulation Decreased Th1 differentiation	(142)
miR-146a/b	Increased in human pulmonary microvascular endothelial cells exposed to TNF	↑ IL-6, IL-8	Increased expression of HSP10	(143)
miR-146b	Decreased in the blood on healthy volunteers infused with LPS	–	Associated with rapid transcriptional activation of IRAK2	(128)
miR-146a-5p	Increased in plasma of sepsis mice (CLP) and sepsis patients	↓ IRAK1	Interacts with TLR7 and activates proteasome Knockout decreases inflammation, improves cardiac function, and survival of sepsis mice	(144)
miR-146a-5p	–	–	Activates TLR7 to induce TNF release, pulmonary inflammation, endothelial barrier disruption and ARDS in sepsis mice	(145)
miR-150	Decreased in MDSCs of sepsis mice (CLP) and in serum of sepsis patients	↓ ARG1	Decreased proliferation and immunosuppressive functions of MDSCs from sepsis mice (CLP)	(146)
miR-150	Decreased in the blood on healthy volunteers infused with LPS	–	Associated with rapid transcriptional activation of IRAK2	(128)
miR-150	Increased in the serum of sepsis mice (CLP)	–	–	(147)
miR-150	Increased in the serum of rats challenged with LPS	–	–	(148)
miR-150	Increased during recovery from LPS-induced injury in mice	↓ EGR2	miR-150^-/-^ mice show increased mortality from LPS and CLP Rescuing miR-150 in lung endothelial cells decreased EGR2-dependent Ang2 expression, restored endothelial barrier function, and reduced mortality	(149)
miR-150	Decreased in the serum of mice challenged with LPS and in sepsis mice (CLP)	↓ NF-κB	Protects HUVECs from LPS-induced apoptosis, decreased TNF and IL-6, ICAM-1, VCAM-1 and E-selectin expression	(150)
miR-150-5p	Decreased in H9c2 cardiomyocytes exposed to LPS	↓ MALAT1	Decreased IL-6 and TNF production	(151)
miR-150-5p	Decreased in the heart of rats challenged with LPS	–	Decreased myocardial apoptosis associated with a reduced expression of Akt2, cleaved caspase 3 and Bax, and increased expression of Bcl-2 in rat heart and H9c2 cardiomyocytes	(152)
miR-150	Decreased in HUVECs exposed to LPS	↓ MALAT1	Decreased TNF and IL-6, ER stress-related proteins, cleaved caspase 3, Bax, apoptosis and increased IL-10 and Bcl-2 in LPS-stimulated HUVECs and PAECs from sepsis mice (CLP)	(153)
miR-150-5p	Decreased in RAW 264.7 macrophages exposed to LPS	↓ Notch1	Inhibits LPS-induced apoptosis and TNF, IL-1β, IL-6 production	(154)
miR-150	Decreased in THP-1 cells exposed to LPS	↓ STAT1	Decreased IL-1β, IL-6 and TNF secretion	(155)
miR-150-5p	Decreased in HK-2 human proximal renal tubular epithelial cells and in mice exposed to LPS	↓ MEKK3	Inhibits LPS-induced JNK pathway, apoptosis, inflammation (IL-1β, IL-6, TNF, BUN, Scr), and outcome of sepsis mice with AKI	(156)
miR-150-5p	Decreased in H9C2 cardiomyocytes and myocardial tissues of mice exposed to LPS	↓ XIST	Decreased c-Fos axis, TXNIP-mediated pyroptosis and sepsis-induced myocardial injury	(157)
miR-155	Comparison of miR-155-deficient and wild-type sepsis mice (CLP)	↑ Neutrophil extracellular traps	Increased neutrophil recruitment Increased sepsis-induced ALI	(158)
miR-155	Increased in pulmonary endothelial cells of sepsis mice and in HUVECs exposed to TNF	↓ Claudin-1	Increased vascular barrier breakdown and sepsis-related capillary leakage	(159)
miR-155	Increased in intestinal tissue of sepsis mice (CLP)	↑ NF-κB	Increased intestinal barrier dysfunction	(160)
miR-155	Increased in plasma and myocardial tissue of sepsis mice and patients	↑ NO, cGMP ↓ Angiotensin type 1 receptor	Increased sepsis-associated cardiovascular dysfunction	(161)
miR-155	Increased in HPMECs exposed to TNF	↑ IL-6, IL-8	Increased HSP10	(143)
miR-155	Increased in myocardial tissue of sepsis mice	↓ JNK phosphorylation, β-arrestin 2	Decreased sepsis-induced myocardial dysfunction	(162)
miR-155	Increased in liver tissue of sepsis mice	↑ JAK/STAT pathway ↓ SOCS1	Increased sepsis-induced AHI	(163)
miR-155	Increased in myocardial tissue of mice exposed to LPS	↓ Pea15a	Increased sepsis-induced myocardial dysfunction	(164)
miR-181-5p	Decreased in kidneys of sepsis mice (CLP)	↓ HMGB1	Decreased inflammatory response Decreased renal and hepatic dysfunction	(165)
miR-181a	Increased in mouse DCs exposed to HMGB1	↓ TNF mRNA	Dual influence of HMGB1 on maturation and cytokine expression in DCs (↑ at low but ↓ at high concentrations)	(166)
miR-181a	Increased in lung tissues of mice exposed to LPS	↓ Bcl-2	Increased apoptosis on ALI by down-regulation of Bcl-2	(167)
miR-181b	Decreased in myocardial tissue of sepsis rats (CLP)	↓ HMGB1	Decreased apoptosis of myocardial cells Decreased sepsis-induced myocardial injury	(168)
miR-181b	Decreased in HUVECs exposed to TNF	↓ NF-κB pathway, VCAM-1, importin-α3	Decreased sepsis-induced vascular inflammation and ALI	(169)
miR-181b	Increased in bone marrow of sepsis mice (CLP)	↑ NFI-A	Increased number of MDSCs by arresting myeloid progenitor differentiation and maturation miR-181b antagomiR improves late-sepsis survival	(86)
miR-181b	High levels in MDSCs from sepsis mice (CLP)	–	miR-181b up-regulated by phospho-STAT3 and C/EBPβ binding to miR-181b promoter in MDSCs, leading to MDSCs expansion	(87)
miR-181	Increased by ouabain in airway epithelial cells A549 and by LPS in THP-1 monocytic cells	↓ TNF mRNA stability	miR-181d agomir increases bacterial burden and decreases survival of sepsis mice (CLP)	(170)
miR-186	–	↓ PTEN ↑ PI3K/ARG (PTEN targets)	miR-186 administration in sepsis rats (CLP) decreases p53 via increased PI3K/AKT in kidney cells and decreases AKI	(171)
miR-186-5p	Decreased in sepsis patients	↓ NAMPT	miR-186-5p inhibited sepsis-induced coagulation disorders via targeting NAMPT and deactivating the NF-κB pathway	(172)
miR-194	Increased in rat H9c2 cardiomyocytes exposed to LPS	↓ Slc7a5 gene ↑ β-catenin, cyclin D1 (hypothesis)	Increased sepsis related myocardial injury	(173)
miR-195	Increased in lung and liver tissues of sepsis mice (abdominal sepsis)	↓ Bcl-2, Sirt1, Pim-1	Increased apoptosis Increased sepsis-induced ALI and AHI	(174)
miR-195-5p	Decreased in LPS-treated cardiomyocytes and sepsis mice (CLP)	↓ ATF6	Decreased inflammation, apoptosis, oxidative stress and endoplasmic reticulum stress in CLP mice	(175)
miR-199a	Increased in intestinal tissues of sepsis mice	↓ Surfactant protein D ↑ NF-κB pathway	Increased apoptosis in epithelial cells of intestinal tissues Increased intestinal barrier dysfunction	(176)
miR-200c-3p	Increased in A549 cells infected with H1N1 or H5N1 influenza virus	↓ ACE2 protein	Increased ALI and ARDS following viral infection (via NF-κB pathway)	(177)
miR-212-3p	Increased in RAW 264.7 macrophages exposed to LPS	↓ HMGB1	Decreased TNF and IL-6 production Decreased p38 and ERK1/2 phosphorylation (via HMGB1 inhibition)	(178)
miR-214-3p	Increased in myocardiac tissues of sepsis mice	↑ p-AKT, p-mTOR ↓ PTEN	Decreased sepsis-induced myocardiac dysfunction Decreased autophagy via AKT/mTOR pathway	(179)
miR-221	Increased in RAW 264.7 mouse macrophages exposed to LPS Sepsis mice (CLP)	↓ JNK2	Increased MCP-1 and CXCL1 levels, lung inflammation and injury in sepsis mice	(180)
miR-223	Increased in lungs of mice exposed to cigarette smoke and LPS and human in pulmonary cells and monocytes exposed to cytokines	↓ HDAC2	miR-223 levels negatively correlate with the HDAC2 expression in lungs from COPD patients Reduced HDAC2 increased fractalkine (CX3CL1) expression	(181)
miR-223	Increased in white blood cells of sepsis patients (especially survivors)	↓ FOXO1	Decreased lymphocytes apoptosis Negative correlation with SOFA score and clinical severity	(182)
miR-223	Decreased in HCAECs exposed to TNF	–	Platelet-derived miR-223 decreased ICAM1 expression in endothelial cells and reduced the binding of PBMCs to HCAECs	(183)
miR-326	Decreased in lung tissues and macrophages of mice exposed to LPS and sepsis mice (CLP)	↓ TLR4	Decreased sepsis-induced ALI	(184)
miR-375	Decreased in whole blood of sepsis patients	↓ miR-21, JAK2, STAT3	Decreased MDSCs in sepsis mice (CLP) miR-375 agomir promotes survival of sepsis mice	(185)
miR-376b	Decreased in renal tubular cells in sepsis mice with AKI	↓ NF-κB inhibitor ζ	Increased sepsis-induced AKI	(186)
miR-494	Increased in human lung cancer cells	↓ NQO1, Nrf2	Increased sepsis-induced ALI	(187)
miR-494-3p	Decreased in plasma of sepsis patients and in RAW 264.7 macrophages	↓ TLR6	Decreased sepsis-induced inflammatory response	(188)
miR-499a	Decreased in HUVECs exposed to LPS	↓ STAT1	Decreased LPS-induced inflammatory injury and apoptosis	(189)
miR-574-5p	Increased in serum of sepsis patients (especially survivors)		Increased viability of renal cell culture line (HK-2) Decreased sepsis-induced AKI	(190)
miR-1184	Decreased in THP-1 cells exposed to LPS and serum of pediatric sepsis patients	↓ TRADD	Decreased expression of TRADD, p65, IL-1β, IL-6 and TNF when overexpressed in THP-1 monocytic cells exposed to LPS	(191)
miR-1184	Decreased in monocytes exposed to LPS and in pediatric sepsis patients	↓ IL-16	Negatively correlates with IL-1β, IL-6, IL-16 and TNF in pediatric sepsis patients Overexpression of IL-16 reverses miR-1184-mediated inhibition of IL-1β, IL-6 and TNF in human monocytes	(192)
miR-1298	Increased in exosomes of sepsis patients	↓ SOCS6	Increased bronchial epithelial cell injury via SOCS6/STAT3 pathway Reverse effects by miR-1298 inhibition	(193)
miR-2055b	Increased in serum and organ tissues (lung, liver, spleen, colon) of sepsis mice	↓ HMGB1	Increased cholinergic anti-inflammatory activity in late sepsis via HMGB1 suppression	(194)

ACE2, angiotensin-converting enzyme 2; AChE, acetylcholinesterase; ADAR1, adenosine deaminase acting on RNA 1; AHI, acute hepatic injury; AKI, acute kidney injury; ALI, acute lung injury; Ang2, angiopoetin-2; ARDS, acute respiratory distress syndrome; ARG1, arginase 1; ATG, autophagy related; BALF, bronchoalveolar lavage fluid; BCL2, B-cell lymphoma 2; BCL2L2, BCL2-like 2; BMDM, bone marrow-derived macrophage; BNIP3L, BCL2 interacting protein 3 Like; BRD4, bromodomain containing 4; CCL, C‐C motif chemokine ligand; BUN, blood urea nitrogen; C/EBP, CCAAT enhancer binding protein; cGMP, cyclic guanosine monophosphate; CIRP, cold-inducible RNA binding protein; CLP, cecal ligation and puncture; COPD, chronic obstructive pulmonary disease; COX, cyclooxygenase; CRNDE, colorectal neoplasia differentially expressed; CXCL, C‐X‐C motif chemokine ligand; DC, dendritic cell; DNMT1, DNA methyltransferase 1; EGR2, early growth response 2; ErbB4, Erb-B2 receptor tyrosine kinase 4; ERK, extracellular signal-regulated kinase; EZH2, Enhancer of zeste homolog 2; FGFR, fibroblast growth factor receptor; FOSL2, fos-like 2; FOXO1, forkhead box O1; GSKJ4, small-molecule inhibitor of JMJD3; HCAEC, human coronary artery endothelial cell; HK2, human kidney 2; HBV, hepatitis B virus; HMGA2, high-mobility group AT-hook 2; HMGB1, high mobility group box 1; HO-1, heme oxygenase-1; HPMEC, human pulmonary microvascular endothelial cell; HSP, heat shock protein; HUVEC, human umbilical endothelial cell; ICAM, intercellular adhesion molecule; IGF1R, insulin like growth factor 1 receptor; IL, interleukin; IL-13αR1, IL-13 receptor α1; iNOS, inducible nitric oxide synthase; IRAK, IL-1 receptor-associated kinase; JAK, janus kinase; JMJD3, jumonji domain-containing protein D3; JNK, c-Jun N-terminal kinase; lncRNA, long non-coding RNA; LPS, lipopolysaccharide; MALAT1, metastasis-associated lung adenocarcinoma transcript 1; MAPK, mitogen-activated protein kinase; MCEMP1, mast cell-expressed membrane protein 1; MDP, muramyl dipeptide; MEF, mouse embryonic fibroblast; MDSC, myeloid-derived suppressor cell; MEKK2, mitogen-activated protein kinase kinase; MERTK, myeloid-epithelial-reproductive tyrosine kinase; MOF, multiple organ failure; MSC, mesenchymal stem cell; mTOR, mammalian target of rapamycin; MyD88, myeloid differentiation primary response 88; NAMPT, nicotinamide phosphoribosyltransferase; NET, neutrophil extracellular trap; NFI-A, nuclear factor I A; NF-κB, nuclear factor kappa B; NLRP3, NOD-, LRR- and pyrin domain-containing protein 3; NO, nitric oxide; NQO1, NAD(P)H quinone oxidoreductase 1; Notch1, notch receptor 1; Nrf2, nuclear factor E2 p45-related factor 2; NRP1, neuropilin 1; PAEC, pulmonary arterial endothelial cell; PBMC, peripheral blood mononuclear cell; PCT, procalcitonin; PDK1, phosphoinositide-dependent protein kinase 1; PD-L1, programmed death-ligand 1; PI3K, phosphoinositide 3-kinase; PLK1, Polo-like kinase 1; PRKCϵ, protein kinase C epsilon; PTEN, phosphatase and tensin homologous protein; RIP2, receptor-interacting protein kinase 2; Scr, serum creatinine; SIRT1, silent information regulator T1; SLEP, P-selectin; SMAD2, Sma- and mad-related protein 2; SMRT, silencing mediator for retinoid and thyroid hormone receptor; SOCS, suppressor of cytokine signaling; SOFA, sequential organ failure assessment; STAT, signal transducer and activator of transcription; TAK1, transforming growth factor activated kinase 1; TCMK-1, transformed C3H mouse kidney-1; TGFBR2, transforming growth factor beta receptor II; TLR, Toll-like receptor; TNF, tumor necrosis factor; TNIP2, TNFAIP3 interacting protein 2; TOP2A, topoisomerase II alpha; TRADD, TNF receptor type 1-associated DEATH domain protein; TRAF6, TNF receptor-associated factor 6; TRAIL, TNF related apoptosis-inducing ligand; TUG1, taurine-upregulated gene 1; TXNIP, thioredoxin-interacting protein; XIST, X-inactive specific transcript; VCAM-1, vascular cell adhesion molecule 1. ↑ means upregulated, and ↓ means downregulated.

**Table 2:** miRNAs as biomarkers in human sepsis.

miRNA	Subjects (sepsis/controls [n] unless detailed) Sample type	Observations	Reference
miRNome (RNA-Seq)	Adults (117 sepsis survivors and 97 sepsis non-survivors based on 28-day mortality) Serum	Less than 200 miRNAs detected by sequencing **Increased** miR-15a, miR122, miR-193b*, miR483-5p & **decreased** miR-16 ,miR-223 in non-survivors vs survivors miR-15a, miR-16, miR-193b and miR-483-5p are associated with death in logistic regression analysis	(195)
miRNome (RNA-seq)	Adults (21 severe & 8 non-severe sepsis, 23 severe & 21 non-severe non-infective SIRS, 16 no SIRS) Plasma	116 detectable blood miRNAs generally up-regulated in SIRS vs no-SIRS patients and higher in non-infective SIRS than sepsis. Inversely correlate with IL-1, IL-6, IL-8 and CRP levels Top 5 miRNAs (miR-23a-5p, miR-26a-5p, miR-30a-5p, miR-30d-5p & miR-192-5p) discriminate severe sepsis from severe SIRS miRNA levels inversely correlate with IL-1, IL-6, IL-8, CRP, PSP levels, but not SOFA score	(196)
miRNome (RNA-seq)	Adults (22/23) Cells, serum & serum exosomes	77 miRNAs **decreased** & 103 miRNAs **increased** in patients (all compartments) 11 miRNAs in at least one compartment correlate with disease severity **Increased** cellular miR-199b-5p, potential early indicator for sepsis and septic shock **Decreased** exosomal miR-30a-5p & miR-125b-5p, and serum miR-193a-5p in non-survivors	(197)
miRNome (microarray, 3’100 probes)	Adults (6 sepsis with AKI, 6 sepsis without AKI, 3 healthy controls) Serum	37 miRNAs differentially expressed among the groups **Increased** miR-3165, miR-4270, miR-4321 & **decreased** miR-22-3p, miR-23a-3p, miR-142-5p, miR-191-5p, miR-4456 in sepsis vs controls miR-4321 upregulated in sepsis with AKI vs sepsis without AKI	(198)
miRNome (microarray, 2’661 probes)	Adults(31 pneumonia, 34 sepsis secondary to pneumonia, 21 healthy controls) Plasma	**Decreased** miR-940 & **increased** miR-765, miR-4800-5p, miR-6510-5p, miR-6740-5p, miR-7110-5p (microarray on 5 pneumonia & 5 sepsis) **Increased** miR-223-3p & miR-7110-5p in sepsis secondary to pneumonia compared to pneumonia and control groups	(199)
miRNome (microarray, 2’578 miRNAs)	Neonates (36 NEC & 101 sepsis patients, 164 controls) Plasma	16 miRNAs **decreased** & 230 miRNAs **increased** and in NEC vs non-NEC (microarray) **Increased** miR-1290 in NEC vs sepsis and controls 7 of 36 infants with NEC diagnosed 13.3 hours earlier using miR-1290 measurement	(200)
miRNome (microarray)	Adults (60/30) Blood	11 differentially expressed miRNAs **Increased** miR-155 confirmed by RT-qPCR miR-155 positively correlated with SOFA score, predictor of 28-day survival miR-155 levels proportional to of CD39+ Tregs %	(201)
miRNome (TaqMan OpenArray, 754 miRNAs)	Adults. Discovery and independent validation cohort with 530 ARDS patients and critically ill at-risk controls Blood	miR-92a & miR-181a are risk biomarkers for ARDS miR-424 is a protective biomarker for ARDS	(202)
miRNome (TaqMan Open Array, 754 miRNAs)	Adults (21/21) Platelets	121 **decreased** & 61 **increased** in patients **Decreased** miR-26b in patients, associated with increased SELP mRNA expression Lower levels associated with sepsis severity & mortality	(92)
miRNome (microarray, 470 miRNAs)	Adults (17/32) PBMCs and plasma	17 miRNAs differentiate sepsis from controls (microarray) **Decreased** miR-150 & miR-342-5p and **increased** miR-182 & miR-486 in sepsis miR-150 positively correlates with SOFA score & inversely correlates with IL-10, IL-18, TNF	(203)
Microarray (n probes?) & RT-qPCR	Adults (31/34) WBCs and T-cells	35 miRNAs differentially expressed in sepsis vs controls (microarray, 7 patients/group) **Decreased** miR-150, miR-342 & **Increased** miR-15a, miR-16, miR-93, miR-143, miR-223 and miR-424 miR-143 **increased** in T-cells, positive correlation with immunoparalysis miR-150 **decreased** in T-cells, negative correlation with immunoparalysis	(204)
miR-10a	Adults (62/20) PBMCs	**Decreased** in patients Negative correlation with disease severity	(205)
miR-15a miR-15b miR-16 miR-206 miR-223 miR-378 miR-451	Neonates (46/41) Serum	**Increased** miR-15a, miR-16 & **decreased** miR-378 and miR-451 in sepsis neonates	(78)
miR-15a miR-16	Adults (166 sepsis, 32 SIRS, 24 healthy controls) Serum	**Increased** in patients vs controls Higher levels of miR-15a in SIRS than in sepsis patients	(206)
miR-15b miR-122 miR-193b* miR-223 miR-483-5p miR-499-5p	Adults (166/24) Serum	**Increased** miR-223 & **decreased** miR-122, miR-193b*, miR-499-5p in patients	(207)
miR-15a miR-16 miR-122 miR-193b* miR-223 miR-483-5p	Adults(123 on day of admission, and 45 on days 1, 3, 5, 7, 10 and 14 of ICU admission)	**Increased** miR-122 in patients with coagulation abnormalities at days 1, 3, 7 and 10	(208)
miR-15a miR-16	Neonates (32 sepsis/30 controls with respiratory infection/pneumonia) Serum	**Increased** in sepsis neonates	(209)
miR-15a-5p miR-155-5p miR-192-5p miR-423-5p	Adult sepsis patients treated with gentamicin, vancomycin or non-nephrotoxic antibiotics (20/7/19) Blood at day 1, 4 & 7	Minor time-dependent changes of miR-15-5p and miR-423-5p miR-15a-5p at day 7 of gentamicin discriminates AKI and non-AKI miR-155-5p & miR-192-5p positively correlate with creatinine and NGAL in vancomycin miR-192-5p & miR-423-5p positively correlate with PCT and IL-6 in non-nephrotoxic antibiotics	(210)
miR-15b miR-378a	Neonates (25/25) Serum	**Increased** miR-15b in sepsis neonates **Decreased** miR-378a in sepsis neonates	(211)
miR-16a miR-451	Neonates (25/25) Serum	**Increased** in sepsis neonates	(212)
miR-19b-3p	Adults (103/98) Serum	**Decreased** in patients Independent prognostic factor for 28-day survival Negative association with IL-6 and TNF	(213)
miR-21	Adults (219/219) Plasma	**Decreased** in patients Poor predictive value for 28-day mortality risk	(214)
miR-21	Children (88/26) Blood	**Increased** in sepsis child/teens (0.2-19 years old)	(85)
miR-21 miR-29a miR-31 miR-146a miR-155	Neonates (42/42) Plasma	**Decreased** miR-29a & miR-146a & **increased** miR-21 & miR-155 in patients (late onset neonatal sepsis) Lower levels of miR-146a in the non-survivors	(215)
miR-22-3p	Adults (69/89) Serum and urine	Negative correlation with acute kidney injury (AKI)	(216)
miR-23a	Adults (27 sepsis, 22 non-infectious SIRS)	**Decreased** in sepsis vs SIRS patients	(89)
miR-23b	Neonates (27 early onset & 21 late onset sepsis) Serum	**Increased** in neonates with early onset sepsis	(217)
miR-25	Adults (70/30) Serum	**Decreased** in patients Negative correlation with severity	(218)
miR-26b	Adults (68 AKI and 87 non-AKI sepsis patients and 57 patients with non-infectious SIRS) Urine	**Increased** in patients with sepsis-associated AKI	(219)
miR-34a miR-199a-3p	Neonates (90/90) Serum	**Decreased** in sepsis neonates Associated with severity	(220)
miR-96	Neonates (66/58*) Serum	**Decreased** in sepsis neonates Targets IL-16	(221)
miR-101-3p	Neonates -	**Increased** in sepsis neonates Associated with PCT, CRP, IL-8 and TNF levels	(222)
miR-103	Adults (108/89) Serum	**Decreased** in patients Negative correlation with IL-1β, IL-6 and TNF levels	(223)
miR-103 miR-107	Adults (196/196) Plasma	**Decreased** in patients **Decreased** in ARDS when compared to non-ARDS patients Negative correlation with 28-days mortality	(224)
miR-122	Adults (25/25) Serum	**Decreased** in patients Limited predictive value for determination of outcome	(225)
miR-122	Adults (108/20) Serum	**Increased** in patients Independent risk factor for 30-day mortality	(226)
miR-122	Adults (232/24) Serum	**Decreased** in patients **Decreased** in ARDS when compared to non-ARDS patients Negative correlation with 28-days mortality	(227)
miR-122 miR-133a miR-143 miR-150 miR-155 miR-192 miR-223	Adults (204 ICU patients, among which 127 with sepsis) Serum	**Increased** miR-133a and **decreased** miR-143 & miR-223 in patients who did not survive the ICU stay A combination of 2 and 3 miRNAs predicts patients’ long-term prognosis and survival in ICUs	(228)
miR-124	Adults (82/82) Plasma	**Decreased** in patients Negative correlation with lncRNA NEAT1 Negative correlation with 28-days mortality	(229)
miR-125a	Adults (196/196) Plasma	**Decreased** in patients Negative correlation with lncRNA MALAT1	(230)
miR-125a/b	Adults (150/150) Plasma	**Increased** in patients Positive correlation of miR-125b with 28-days mortality	(231)
miR-125b	Adults (120/120) Plasma	**Increased** in patients Positive correlation with 28-days mortality	(232)
miR-125	Adults (126/125) Plasma	**Decreased** in patients Negatively correlation with Scr, CRP, APACHE II score, SOFA score, TNF, IL-6, IL-8, IL-17	(233)
miR-126	Children (60/46) Serum	**Decreased** in patients Negative correlation with sepsis severity	(234)
miR-126	Adults (208/210) Plasma	**Increased** in patients Positive correlation with 28-days mortality	(235)
miR-127 miR-191 miR-320a	Adults (200 ICU among whom 140 sepsis/100) Platelets	**Increased** miR-320a/miR-127 ratio in sepsis patients	(236)
miR-130b-3p	Adults (15/7) Serum	**Increased** in patients	(62)
miR-132 miR-146a miR-155 miR-223	Neonates (25/25) Plasma	**Decreased** miR-132 & miR-223 in sepsis patients	(237)
miR-132 miR-223	Adults (80 sepsis-induced cardiomyopathy, 60 controls) Serum	Decreased in patients Negative correlation with CK, TNF & IL-6	(238)
miR-133a	Adults (223/76) Serum	**Increased** in patients Positive correlation with sepsis severity and 28-days mortality	(147)
miR-133	Adults (30/30) Serum	**Increased** in patients	(123)
miR-143	Adults (218 critically ill patients among which 135 sepsis/76 healthy controls) Serum	Trend for **decreased** levels in critically ill patients vs healthy controls No correlation with inflammatory markers Negative correlation with 28-days mortality	(239)
miR-143	Adults (103 sepsis, 95 SIRS, 40 healthy controls) Serum	**Increased** in patients, higher in sepsis than in SIRS Correlation with disease severity	(240)
miR-146a miR-155	Adults (146/19) Plasma	**Increased** in severe patients Positive correlation with sepsis-induced ALI	(241)
miR-146a	Adults (50 sepsis, 30 SIRS, 20 healthy controls) Serum	**Decreased** in sepsis vs SIRS patients and healthy controls	(242)
miR-146a	Adults (14/14) Plasma	**Decreased** in patients	(243)
miR-146a	Children (55/60) Serum	**Decreased** in sepsis children	(244)
miR-146a-5p	Adults (11/12) Plasma	**Increased** in patients Positive correlation with lactate and partial thromboplastin time	(144)
miR-146a/b	Adults (180/180) Plasma	**Increased** in patients Positive correlation of miR-146b with 28-days mortality	(245)
miR-146b	Adults (104/100) Plasma	**Decreased** in patients Negative correlation with ARDS	(246)
miR-147b	Adults (130 bacterial sepsis, 69 dengue hemorrhagic fever and 82 healthy controls) Plasma	**Increased** miR-146-3p, miR-147b, miR-155, miR-223 in sepsis compared to hemorrhagic fever and healthy controls Correlation with severity miR-147b diagnostic biomarker for patients with bacterial sepsis and septic shock	(247)
miR-150	Adults (223/76) Serum	No significant difference in critically ill patients with and without sepsis Negative correlation with 28-days mortality	(248)
miR-150 miR-4772-5p	Adults (22 SIRS, 23 sepsis, 21 healthy controls) Blood	**Decreased** miR-150 in patients, more decreased in sepsis vs SIRS patients **Increased** miR-4772-5p in sepsis patients	(249)
miR-150	Adults (120/50) Serum	**Decreased** in patients Negative correlation with IL-6 and TNF serum level, renal function and 28-days mortality	(150)
miR-150 Let-7a	Adults (22/20), urosepsis Leukocytes	**Decreased** in patients	(250)
miR-150	Adults (29 survivors and 12 non-survivors of sepsis) Serum	Most strongly **decreased** miRNA in healthy subjects infused with endotoxin	(251)
miR-150	Adults (30, with AKI/15) Serum	**Decreased** in patients	(156)
miR-150	Adults (78 sepsis/62 non-septic trauma patients/10 healthy controls)	**Decreased** in sepsis patients, increased in non-septic trauma patients	(146)
miR-150	Adults (299 survivors and 138 non-survivors of sepsis) Blood	**Decreased** miR-150 associated with 28-day mortality Combination of miR-150 and SOFA score improved prediction of prognosis	(252)
miR-155	Adults (73/83) Plasma	Positive correlation with sepsis-induced ALI and ARDS	(253)
miR-155	Adults (10/10) BALF	**Increased** in patients (all with ARDS)	(254)
miR-181a	Neonates (102/50) Serum	**Decreased** in sepsis neonates	(255)
miR-186-5p	Adults (34 sepsis and 34 respiratory infection/pneumonia)	**Decreased** in sepsis patients	(172)
miR-206	Adults (93/28) Serum	**Increased** in patients	(256)
miR-218	Adults (53/20) PBMCs and Tregs	**Decreased** in patients **Decreased** according to severity	(257)
miR-223	Adults (187/186) Plasma	**Increased** in patients Positive correlation with APACHE II score, CRP, cytokines Higher in non-survivors than in survivors	(258)
miR-223	Adults (143/44) White blood cells	**Increased** in patients Higher in survivors than in non-survivors Negative correlation with lymphocyte apoptosis	(182)
miR-223	Adults (50 sepsis, 30 SIRS, 20 healthy controls) Serum	**Decreased** in sepsis vs SIRS patients and healthy controls	(242)
miR-223	Adults (137/84) Serum	No differential expression	(259)
miR-223	Adults (122/122)	**Increased** in sepsis Positive correlation with APACHE II and SOFA scores, and 28-day mortality	(260)
miR-328	Adults (110/89) Serum	**Increased** in sepsis patients Positive correlation with Scr, WBC, CRP, PCT, APACHE II score, and SOFA score	(261)
miR-410-3p	Neonates (88 sepsis, 86 pneumonia) Serum	**Decreased** in sepsis versus pneumonia patients Correlation with levels of lncRNA NORAD	(262)
miR-451a	Adults (98/65) Serum	**Increased** in patients Positive correlation with sepsis-induced cardiac dysfunction	(263)
miR-452	Adults (47 sepsis with AKI, 50 sepsis without AKI, 10 healthy controls) Serum and urine	**Increased** in serum and urine of sepsis vs controls. Higher in patients with AKI vs no AKI	(264)
miR-494-3p	- Blood	**Decreased** in sepsis patients Downregulates TLR6	(188)
miR-495	Adults (105/100) Serum	**Decreased** in patients **Decreased** in septic shock when compared to non-septic shock patients Negative correlation with sepsis-induced cardiac dysfunction	(265)
miR-1184	Children (30/30) Serum	**Decreased** in sepsis children	(191)
miR-1184	Neonates (72/56) Serum	**Decreased** in sepsis neonates Negative correlation with IL-16	(192)

*Controls were neonates with respiratory infection or pneumonia.

AKI, acute kidney injury; ALI, acute lung injury; APACHE, acute physiology and chronic health evaluation; BALF, bronchoalveolar lavage fluid; CK, creatine kinase; CRP, C reactive protein; lncRNA, long noncoding RNA; NEAT1, nuclear enriched abundant transcript 1; NEC, necrotizing enterocolitis; NGAL, neutrophil gelatinase-associated lipokalin; PCT, procalcitonin; PSP, pancreatic stone protein; Scr, serum creatinine; SELP, P-selectin; SIRS, systemic inflammatory response syndrome; SOFA, sequential organ failure assessment; TLR, Toll like receptor; Treg, regulatory T cells; WBC, white blood cell.

### 3.1 miRNAs and sepsis pathophysiology

#### 3.1.1 miRNAs and innate immune cells

First, it should be recalled that miRNAs are not acting only as brakes, but also as promoters of inflammatory and innate immune responses. Cues to how miRNAs weight the inflammatory response have been obtained in studies using Dicer 1-deficient mouse macrophages depleted of miRNAs. Contrary to expectations, Dicer 1-deficient macrophages produce reduced levels of tumor necrosis factor (TNF), IL-6 and IL-12 in response to TLR1/2, TLR4, and TLR9 stimulation (84). It has been proposed that miRNAs expressed constitutively repress innate immune genes to preserve homeostasis, while stimulus-induced miRNAs fine-tune inflammatory responses and return to homeostasis (266).

miRNAs modulate immune signals by targeting positive or negative players of immune signaling pathways. This process is highly dynamic for several reasons. First, miRNAs are differentially expressed in innate and non-innate immune cell types. Second, miRNA expression is upregulated or downregulated in response to MAMPs/DAMPs/cytokines, and subjected to circadian rhythm (267). For example, miR-146a and miR-155 are upregulated while miR-27a and miR-532-5p are downregulated in macrophages exposed to LPS. Third, miRNAs regulate their own expression. The proinflammatory miR-375 inhibits the expression of the anti-inflammatory miR-21 by targeting the Janus kinase (JAK) 2-signal transducer and activator of transcription protein (STAT) 3 signaling pathway (185). Fourth, one miRNA targets many mRNAs, and one mRNA is regulated by various miRNAs. Consequently, miRNAs have additive or antagonistic effects on their targets. Fifth, one miRNA either inhibits or activates immune signaling, participating to feedback loop mechanisms controlling gene expression. Sixth, miRNAs circulate in fluids and act at a distance (268–272).

miRNAs target transcription factors, signaling proteins and growth factors to influence hematopoiesis and modulate the development of innate and adaptive immune cells. miRNAs regulate the functions of mature innate immune cells, including migration, phagocytosis, efferocytosis, production of cytokines, tolerance, tissue remodeling and promotion of tumor development (273–278). Macrophages display a continuum of functional states, ranging from proinflammatory M1 macrophages to pro-resolving/anti-inflammatory M2 macrophages. miR-155 is up-regulated in M1 macrophages. The knockout of *mir155* and miR-155 antagomir reduces the expression of *Inos*, *Il1b*, *Il6*, *Il12*, and *Tnf* in M1 macrophages. In fact, around half of the 650 genes that make up the M1 signature rely on miR-155 (279). miR-130b-3p inhibits IRF1 expression, M1 macrophage polarization and the production of C‐C motif chemokine ligand 5 (CCL5), C‐X‐C motif chemokine ligand 10 (CXCL10), inducible nitric oxide (NO) synthase (iNOS) and TNF (63). miR-223 regulates peroxisome proliferator-activated receptor-γ mediated M2 macrophage activation (280). Overall, many miRNAs have been associated with the polarization/activity of M1 macrophages (miR-9, miR-26a-2, miR-125a-3p, miR-125b, miR-127, miR-155-5p, miR-181a, miR-204-5p, miR-451) and M2 macrophages (miR-27a, miR-29b-1, miR-34a, miR-124, miR-125a-5p, miR-132, miR-143-3p, miR-145-5p miR-146a-3p, miR-193b, miR-222, miR-223, let-7c) (278, 281–283). miRNAs influence the differentiation, expansion and biological activities of myeloid-derived suppressor cells (MDSCs) that are associated with sepsis morbidity and mortality (17, 28, 284, 285). Thus, miRNAs shape both inflammation-associated antimicrobial defenses, anti-inflammatory and pro-resolving immune reactions and immunosuppression. The two facets can be driven by a single miRNA entity. miR-466l expression in polymorphonuclear neutrophils (PMNs) induces inflammation and precedes miR-466l expression in macrophages acquiring pre-resolving functions (286).

#### 3.1.2 miRNAs and endothelium and coagulation activation in sepsis

DAMPs and MAMPs released during sepsis activate the complement and coagulation systems. Disseminated intravascular coagulation (DIC) affects around 35% of sepsis patients. Beside thrombosis, DIC is associated with bleeding due to the consumption of clotting factors, anticoagulant proteins, and platelets (9, 11). Thrombocytopenia develops in about 50% of sepsis patients. Signaling through PRRs and cytokine receptors triggers endothelial cells, increasing the expression of adhesion molecules, vascular permeability, transcellular migration, microcirculation lesions, tissue ischemia and organ failure (287, 288). Many endogenous and microvesicles-derived miRNAs regulate endothelial cell functions acting on apoptosis, proliferation, migration and inflammation (289–292). For example, miR-155 is increased in pulmonary endothelial cells of sepsis mice, targets the tight junction protein Claudin-1 and induces capillary leakage during infection (159). In a model of ALI, endothelial cell-derived exosomal miRNA-125b-5p downregulates topoisomerase II α resulting in reduced lung injury and inflammatory cell infiltration in the pulmonary mesenchyme (112). Decreased exosomal miR-125b (and miR-30a-5p) is associated with mortality in sepsis patients (197).

Platelets play a role beyond thrombosis and hemostasis, regulating innate immune cells including PMNs, monocytes and macrophages (287, 293, 294). Platelets are an important sources of miRNAs that are released through microvesicles or exosomes and are taken up by endothelial cells and macrophages (113, 295). Reduced miR-26b in platelets is associated with increased P-selectin expression, and with severity and mortality in sepsis patients (92). In fact, miR-26b reduces platelet adhesion and aggregation in mice (296). An increased miR-320a/miR-127 ratio in platelets could help detecting sepsis (236). Platelet microvesicles containing miR-126-3p are taken up by macrophages, strongly affecting the transcriptome and decreasing the expression of cytokines/chemokines/growth factors in the cells (113). Additionaly, miR-126-3p is associated with platelet activation (297). miR15b-5p and miR-378a-3p in platelet-derived exosomes obtained from sepsis patients induce the formation of neutrophil extracellular traps (NETs) involved in organ injury (80). On the contrary, platelet microparticles containing miR-223 reduce intercellular adhesion molecule 1 (ICAM-1) expression and binding to peripheral blood mononuclear cells by endothelial cells, providing a possible protective role against excessive sepsis-induced vascular inflammation (183).

#### 3.1.3 miRNAs and host response to endotoxin (LPS)

**Figure 3:**
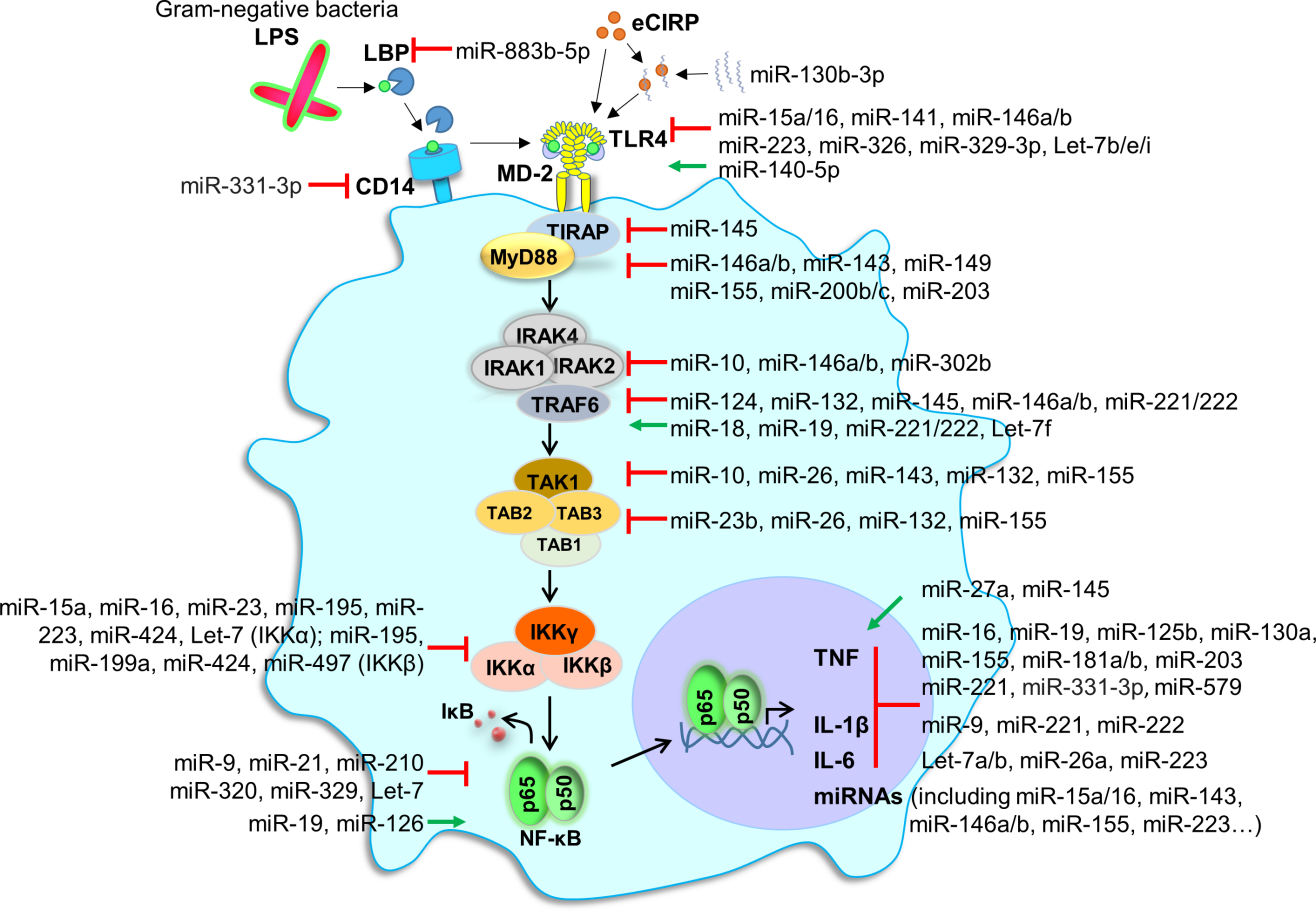
miRNAs and endotoxin sensing and signaling. The figure shows the recognition and intracellular signaling events following the sensing by monocytic cells of LPS from Gram-negative bacteria. LPS aggregates are dissociated by the LPS-binding protein (LBP). LPS/LBP complexes are transferred to CD14, a glycosylphosphatidylinositol-anchored molecule expressed on the membrane of monocytic cells. CD14 transfers LPS to TLR4 together with MD-2. This induces the recruitment of TIR domain-containing adaptor protein (TIRAP) and myeloid differentiation primary response gene (MyD88). MyD88 is involved in early nuclear factor-κB (NF-κB) activation and pro-inflammatory gene expression. NF-κB signaling is involved in the expression of many miRNAs. For reasons of simplicity, we did not depict the TIR domain-containing adaptor inducing IFNβ (TRIF)-dependent, MyD88 independent, pathway involved in IRF signaling and late NF-κB activation. Red lines depict inhibition, while green lines depict activation (by miRNAs). eCIRP, extracellular cold-inducible RNA binding protein; IκB, inhibitory kappa B; IKK, IκB kinase; IRAK, IL-1 receptor-associated kinase-1; TAB, transforming growth factor-β (TGF-β) activated kinase 1; TRAF, TNF receptor-associated factor; TAK1, TGF-β activated kinase-1.

Many studies analyzed miRNAs selected based on prior knowledge or miRNA screenings. While very instructive on a case-by-case basis, reductionist explorations tackle a small part of the role of miRNAs. A good illustration comes from reports on endotoxin, which is used as a model system to study host response to Gram-negative bacteria (**Figure 3**). The sensing of extracellular LPS by innate immune cells involves LPS binding protein (LBP), CD14, MD-2 and TLR4 (298, 299). TLR4, anchored at the cell membrane, recruits the adaptor molecule myeloid differentiation primary response 88 protein (MyD88). MyD88 activates a cascade of phosphorylation initiated at the level of IL-1 receptor (IL-1R)-associated kinase-1 (IRAK1) and TNF receptor-associated factor 6 (TRAF6), filling the NF-κB, IRFs and MAPK signaling pathways. These pathways control the transcription of immune response genes. Note that TLR4 shuttling to late endosome induces an alternative signaling through the adaptor molecule TIR domain-containing adaptor inducing IFNβ (TRIF). TRIF initiates IRF3 and late NF-κB activation, involved in the production of type I IFNs and IFN-inducible genes. For reasons of simplicity, this pathway is not described on **Figure 3**.

A few dozen of miRNAs, among which miR-15a, miR-16, miR-17-5p, miR-21, miR-25, miR-31, miR-98, miR-124-5p, miR-125b, miR-140-5p, miR-141, miR-146a, miR-149-5p, miR-155 miR-181c, miR-203-5p, miR-221, miR-326, miR-378, miR-448 and miR-466I, interfere at different levels with LPS sensing and LPS-induced signaling pathways (**Figure 3**). Note that miR-15a/16, miR-17-5p, miR-25, miR-125b, miR-141, miR-326 and miR-448 inhibit TLR4 expression, while miR-140-5p increases TLR4 expression. In addition, dozens of miRNAs among which miR-9-5p, miR-19a-5p, miR-21, miR-29, miR-93, miR-98, miR-125, miR-221, miR-222, miR-223 and let-7a-5p target the expression of downstream proinflammatory and anti-inflammatory cytokines. Finally, the inflammatory response itself regulates the expression of proinflammatory and anti-inflammatory miRNAs (273–277, 300–302). These observations provide insight into the complexity of miRNAs interactions during host antimicrobial responses, and highlight the challenge of taking a comprehensive and integrated view of the impact of miRNAs on immune responses.

#### 3.1.4 miRNAs and endotoxin tolerance

Exposure of isolated innate immune cells or whole body to low amounts of LPS induces a transient period of refractory response to subsequent exposure to LPS, generally attested by inhibition of cytokine production. This phenomenon is known as endotoxin tolerance. Expression studies suggest that miR-146a and miR-146b are involved in endotoxin tolerance in THP-1 human monocytic cells (303–305). miR-146a disrupts both transcription and translation of *TNF* gene in tolerant THP-1 cells (305). miR-146b is induced by the anti-inflammatory cytokines IL-10 and transforming growth factor (TGF)-β, but repressed by IFNγ which reverses endotoxin tolerance (304). Tolerance extends beyond LPS and TLR4 signaling. Bacterial lipoproteins recognized through TLR2 increase miR-146a expression and render THP-1 cells hypo-responsive to subsequent stimulation by *Salmonella typhimurium*. This is associated with a strong reduction of IRAK-1, phosphorylated inhibitory kappa B α (IκBα), and TNF production in tolerant THP-1 cells (306). Epigenetic mechanisms are involved in the establishment of tolerance. miR-146a and miR-155 are co-regulated in naïve and tolerant RAW 264.7 mouse macrophages. LPS stimulation induces histone 3 lysine 4 trimethylation (H_3_K_4_me_3_, a mark of transcriptionally active genes) and NF-κB p65 binding to miR-146a and miR-155 gene loci. The induction of tolerance is associated with a shift towards repressive H_3_K_9_me_3_ mark and the recruitment of CCAAT/enhancer-binding protein (C/EBP) β and p50 inhibitory component of the NF-κB complex to miR-146a and miR-155 genes (307).

### 3.2 Examples of miRNAs studied as modulators of innate immune responses and biomarkers of sepsis

**Table 1** summarizes observations about miRNAs obtained in cells exposed to MAMPs/DAMPs, and in animals and humans with sepsis. **Table 2** summarizes observations about miRNAs as potential biomarkers of human sepsis. We will not describe all studies because it would be tedious if not impossible. We will focus on miR-15a, miR-16, miR-122, miR-143, miR-146a/b, miR-150, miR-155 and miR-223 taken as examples of important and versatile miRNAs, and because these miRNAs are discussed in several publications in the sepsis field. This selection is arbitrary, but we will nevertheless see that even a limited sample of miRNAs provides insight into the complexity by which miRNAs on impact sepsis. Observations reported in precedent chapters will not be repeated.

#### 3.2.1 miR-15a/16

miR-15a and miR-16 are members of the miR-15 family comprising miR-15a, miR-15b, miR-16-1, miR-16-2, miR-195, and miR-497. *miR-15a/16-1* cluster resides on human chromosome 13. miR-15a and miR-16 share the same seed sequence suggesting that they mediate similar biological functions.

##### 3.2.1.1. Anti-inflammatory activity

miR-15a and miR-16 are commonly viewed as anti-inflammatory miRNAs. Bacterial infection and LPS increase miR-15a/16 in mouse bone-marrow derived macrophages, and in mouse lungs. miR-15a/16 target TLR4 and IRAK-1 in RAW 264.7 mouse macrophages exposed to LPS (78). In agreement, miR-15a/16 deficiency increases the expression of TLR4 through PU.1 (a transcription factor essential for TLR4 expression (308), and the phagocytosis and killing of *E. coli* by macrophages (77). Accordingly, miR-15a/16 knockout mice are resistant to CLP, *E. coli* and LPS-induced lethal sepsis (77). As an example of the connection between ncRNAs, the lncRNA SNHG16 downregulates the expression of miR-15a/16 and counter-regulates the inhibitory effects of miR-15a/16 on the expression of TLR4 in RAW 264.7 macrophages (209).

##### 3.2.1.2 Inflammatory activity

LPS increases miR-16 expression in human monocytic cells and biliary epithelial cells through the MAPK pathway. In a counter-regulatory manner, miR-16 suppresses silencing mediator for retinoid and thyroid hormone receptor, and increases NF-κB transcriptional activity and expression of IL-1α, IL-6 and IL-8 in LPS-stimulated cells (81). Similarly, miR−15a−5p is increased in RAW 264.7 macrophages exposed to LPS, targets TNF-induced protein 3−interacting protein 2, activates the NF-κB pathway and increases cytokine production (79). A miR-15a-5p inhibitor reduces IL-1β, IL-6 and TNF and inflammatory response in mice challenged with LPS (79).

##### 3.2.1.3 Biomarker value

miR-15a and miR-16 are increased in patients with systemic inflammatory response syndrome (SIRS) and sepsis patients when compared to healthy controls (n = 66, 32, 24). miR-15a levels are higher in SIRS than in sepsis patients (206). The screening of 13 miRNAs (miR-15a, miR-16, miR-21, miR-27a, miR-34a, miR-126, miR-150, miR-155 miR-181b, miR-223, miR-125b, miR-146a, miR-486) in 62 adult sepsis patients and 32 healthy controls shows that miR-15a, miR-16, miR-21, miR-125b, miR-126, miR-146a, miR-155, miR-181b, miR-223 are increased in sepsis patients. miR-15a is lower in patients with shock than in patients without shock (309). In a prospective observational study (117 survivors and 97 non-survivors with sepsis), a miRNome analysis shows that miR-15a (together with miR122, miR-193b* and miR483-5p) is increased in sepsis non-survivors, while miR-16 (and miR-223) is decreased (195).

Among seven miRNAs (miR-15a, miR-15b, miR-16, miR-206, miR-223, miR-378 and miR-451) measured in 46 neonatal sepsis patients, only miR-15a and miR-16 are increased, while miR-378 and miR-451 are decreased. Receiver operating characteristic (ROC) curve analyses suggest that miR-15a and miR-16 serum levels are good predictors of neonatal sepsis with area under the curves (AUCs) of 0.85 and 0.87 (78). miR-15a and miR-16 are increased in the serum of neonates with sepsis when compared to neonates with respiratory infection or pneumonia without sepsis (n = 62 and 32) (309). Finally, two recent studies report increased miR-15b and miR-16a in small cohorts of sepsis neonates (25 sepsis and 25 controls) (211, 212).

Overall, miR-15a/16 drive anti-inflammatory or inflammatory action, and are commonly increased in sepsis patients. A link with disease severity seems more uncertain.

#### 3.2.2 miR-122

miR-122 was identified 20 years ago as a liver specific miRNA in mice (310). miR-122 is encoded on chromosome 18 in humans, and has no close paralog. miR-122 has been especially studied in the context of host response to liver-tropic viruses.

##### 3.2.2.1 Anti-inflammatory activity

miR-122 is decreased in the liver of patients with hepatocellular carcinoma (HCC). The upregulation of miR-122 in HepG2 human hepatocellular carcinoma cell lines inhibits TLR4 expression. Moreover, miR-122 decreases the proliferation and the production of TNF and IL-6 by HepG2 and Huh7 hepatocellular carcinoma cell lines (107).

##### 3.2.2.2 Inflammatory activity

miR-122 targets suppressor of cytokine signaling protein (SOCS) 1 and SOCS3, inducing IFNα/β expression and decreasing hepatitis B virus (HBV) replication (103, 104). miR-122 targets the receptor tyrosine kinases (RTKs) insulin like growth factor 1 receptor (IGF1R), fibroblast growth factor receptor and myeloid-epithelial-reproductive tyrosine kinase. Then, miR-122 decreases STAT3 phosphorylation and increases IRF1 signaling and the expression of IFNs in response to hepatitis C virus (HCV) and the synthetic analog of doubled stranded RNA poly(I:C) (105). miR-122 targets heme oxygenase-1 and decreases HBV expression in hepatoma cells (106). Related to sepsis, miR-122-5p is increased in the heart of rats and in H9c2 rat cardiomyocytes challenged with LPS. Inhibition of miR-122-5p reduces myocardial injury through inhibition of inflammation, oxidative stress and apoptosis in endotoxemic rats (108).

##### 3.2.2.3 Biomarker value

At least four studies have reported decreased miR-122 levels in patients with sepsis when compared to healthy controls (201, 207, 225, 227). miR-122 levels are lower in ARDS than in non-ARDS patients and show a negative correlation with 28-days mortality (227). In contrast with these observations, miR-122 is increased in sepsis patients and is an independent risk factor for 30-day mortality (195, 226). Moreover, the levels of miR-122 (but not miR-15a, miR-16, miR-193b*, miR-223 and miR-483-5p) are higher in patients with coagulation abnormalities than in patients with normal coagulation tested at days 1, 3, 7 and 10 of ICU admission (208). Finally, other studies do not point to miR-122 differential expression in sepsis patients and healthy controls (198, 228). Hence, the biomarker value of miR-122 remains questionable.

#### 3.2.3 miR-143

miR-143 is encoded in a bicistronic locus with miR-145, but has no homology with miR-145. miR-143 is considered as an anti-inflammatory miRNA. Few studies looked at the mechanisms of action of miR-143 in the context of innate immune response and sepsis.

##### 3.2.3.1 Anti-inflammatory activity

The quantification of 455 miRNAs in blood leukocytes from heathy volunteers infused 4 hours with endotoxin identified miR-143 as the only upregulated miRNA. High levels of miR-143 are linked to decreased expression of B-cell CLL/lymphoma 2, a regulator of apoptosis an innate immune signaling, and the silencing of inflammation-related target genes (128). Mycobacterial cell wall glycolipid (Ac2PIM) and muramyl dipeptide (MDP) are recognized by TLR2 and NOD2. In mouse macrophages, Ac2PIM induces miR-143. In turn, miR-143 targets the NOD2 signaling adaptors TGF-β activated kinase-1 (TAK1) and receptor-interacting protein kinase 2. miR-143 suppresses PI3K/PKCδ/MAPK/β-catenin-mediated expression of cyclooxygenase-2 (COX-2), SOCS3 and matrix metalloproteinase (MMP)-9 induced by MDP (129). Thus, miR-143 negatively regulates the NOD2 pathway, which may have consequences on the development of vaccines and Gram-positive bacteria sepsis.

miR-143 is the most significantly downregulated miRNA in nasal mucosal tissues from patients with allergic rhinitis (311). miR-143 dampens inflammatory responses in upper airways (130). Bronchial epithelium cells exposed to angiotensin II (AngII) and LPS increase miR-143 which targets angiotensin converting enzyme 2 (ACE2). A miR-143-3p inhibitor increases ACE2 and decreases inflammatory cytokines and apoptosis in cells exposed to AngII and LPS (132). ACE2 protects mice from ALI induced by sepsis (312), so miR-143 may be used to decrease lung inflammation involved in ARDS. In a mouse model of mycoplasma pneumonia, a miR-143-3p mimic reduces IL-2 and TNF, increases IL-10 and reduces alveolar epithelial cell apoptosis. A miR-143 mimic decreases TLR4, MyD88 and phosphorylated NF-κB p50 in lungs. miR-143 might be used to inhibit the TLR4/MyD88/NF-κB signaling pathway and normalize pulmonary inflammation during pneumonia (133).

Mesenchymal stem/stromal cells (MSCs) therapy improves sepsis outcome. Treating human umbilical cord MSCs with poly(I:C) decreases miR-143 and increases the anti-inflammatory power of MSCs on macrophages. miR-143 targets TAK1 involved in TLR3 signaling and COX-2. The infusion of poly(I:C)-activated MSCs improves survival of CLP mice, while the co-delivery of miR-143 reduces the survival benefit provided by MSCs (134). Targeting miR-143 might have therapeutic potential in dampening inflammatory responses in sepsis. No study reported inflammatory activity of miR-143.

##### 3.2.3.2 Biomarker value

Microarray and RT-qPCR analyses have been used to explore miRNAs in T cells and whole blood in 34 healthy controls and 31 sepsis patients. Thirty five miRNAs are differentially regulated in sepsis patients. miR-143 (and miR-15a, miR-16, miR-93, miR-223 and miR-424) is increased in sepsis patients. miR-143 levels correlate with T cell immuno-paralysis. The discriminatory power of miR-143 in T cells performs well, with an AUC of 0.95. miR-143 correlates positively with sequential organ failure assessment (SOFA; a clinical score based on the assessment of 6 variables representing an organ system: respiration, coagulation, liver, cardiovascular, central nervous system, renal) score (204). Another study reports higher blood levels of miR-143 in patients with sepsis than in patients with SIRS, and in SIRS patients than in healthy controls (n = 103/95/40). miR-143 levels correlate with disease severity, evaluated by SOFA and Acute Physiology And Chronic Health Evaluation (APACHE) II (a clinical score that estimates ICU mortality based on laboratory values, age and previous health conditions) scores (240).

In a prospective observational study, miR-143 is similarly expressed in sepsis survivors and non-survivors (n = 117/97) (195). In a cohort of 218 critically ill patients, among which 135 sepsis patients, miR-143 levels are similar to those measured in healthy controls (n = 76). In ICU patients, miR-143 levels do not correlate with inflammatory markers, but correlate with indicators of organ failure (239).

Contrary to the above, miR-143 serum levels are higher in sepsis survivors than in sepsis non-survivors. The performance of miR-143 is rather modest (AUC = 0.628), yet it is higher than that of C-reactive protein (CRP), leukocyte count, creatinine and international normalization ratio value (239). In a subsequent report, the same team analyzed the prognostic scoring of combinations of miR-143 (and miR-122, miR-133a, miR-150, miR-155, miR-192, miR-223) in 204 ICU patients of whom 127 with sepsis (228). A “3 miRNAs” score based on higher miR-133a or lower miR-143 and miR-223 levels predicts patient survival in ICU. A “2 miRNAs” score (higher miR-133a and lower miR-150 levels) predicts patient long-term prognosis. The predictive power of the scores is increased by adding age into the calculation.

Overall, miR-143 is consensually anti-inflammatory. It is almost invariably increased in sepsis patients. Its usage as a biomarker remains unsure. miR-143 might be valuable incorporated in combined scores, but this should be confirmed in independent studies.

#### 3.2.4 miR-146a/b

miR-146a and miR-146b are encoded on human chromosomes 5 and 10, respectively. They have nearly identical sequences and might share targets (313).

##### 3.2.4.1 Anti-inflammatory activity

The group of David Baltimore reported in 2006 the negative impact of miR-146a/b on signaling in innate immune cells (314). miR-146a/b is an immediate early-response NF-κB-dependent gene induced by microbial components and proinflammatory mediators. IRAK1 and TRAF6 are targets of miR-146a/b (314). Macrophages from *miR-146a* knockout mice are hyper-responsive to LPS, and miR-146a restrains inflammation, myeloid cell proliferation, and oncogenic transformation *in vivo* (315). miR-146a inhibits NF-κB signaling and expression of cytokines, ICAM-1 and E-selectin, and trafficking induced by MAMPs in monocytes, macrophages, DCs, endothelial cells and keratinocytes (137, 138, 159, 316, 317). miR-146a inhibits the expression of STAT1, IFNγ and TNF, and the cytotoxicity of natural killer cells (318). A miR-146a agomir (a synthetic chemically modified double-strand miRNA) inhibits macrophage inflammatory response and protects mice from LPS-mediated organ damage (141). The delivery of a miR-146a-expressing plasmid decreases inflammatory cytokines and organ injury, and increases survival of mice subjected to CLP (138).

##### 3.2.4.2 Inflammatory activity

Exogenous single stranded miR-146a-5p induces inflammatory responses through activation of TLR7 and proteasome, and downregulation of IRAK-1. miR-146a knockout mice show reduced inflammation and organ injury, improved cardiac function, and increased survival to acute sepsis induced by CLP (144). miR-146a-5p-mediated activation of TLR7 induces TNF, pulmonary inflammation, endothelial barrier disruption and ARDS in sepsis mice (145).

##### 3.2.4.3 Biomarker value

miR-146a is increased in the blood of healthy subjects infused with endotoxin (251). Among 7 miRNAs measured in the serum of healthy controls, SIRS patients, and sepsis patients (n = 20/30/50), miR-146a and miR-223 are lower in sepsis patients (AUC = 0.804 and 0.858) (242). Similarly, reduced miR-146a levels discriminate sepsis from SIRS patients (AUC = 0.813) (243). In a pediatric study (n = 60/55 healthy and sepsis patients), miR-146a is decreased in blood and negatively correlated with the levels of C-reactive protein, procalcitonin (PCT), IL-6 and TNF. miR-146a levels correlate with sepsis severity and mortality, showing lower levels of miR-146a in non‐surviving than in surviving patients (244). However, another study does not report differential expression of miR-146a in newborns with or without early-onset sepsis (n = 25/group) (237).

In contrast, miR-146a is increased in two studies analyzing adult patients (241, 245). In the first study (19 healthy controls, 102 sepsis, 44 severe sepsis), the AUCs of miR-146a and miR-155 for predicting 30-day mortality in ALI patients are 0.733 and 0.782 (241). In the second study (180 healthy controls, 180 sepsis patients), miR-146a and miR-146b expression levels are predictors of sepsis risk (AUC = 0.774 and 0.897) (245). miR-146a and miR-146b positively correlate with APACHE II score, SOFA score, creatinine, CRP, IL-1β, IL-6, IL-17 and TNF. miR-146a and miR-146b are higher in survivors than in 28-day non-survivors. miR-146b has a better predictive value than miR-146a (AUC = 0.703 *vs* 0.599).

Overall, miR-146a is traditionally considered as anti-inflammatory, but 2 recent studies seem to contradict the uniform view. In the same manner, it remains unclear how miR-146a/b are modulated in human sepsis.

#### 3.2.5 miR-150

miR-150 is encoded on human chromosome 19. miR-150 plays a role in hematopoiesis (319). miR-150 affects apoptosis, maturation and differentiation of lymphocytes and NK cells, and autoimmune diseases (320, 321). miR-150 is one of the four miRNAs (with miR-146b, miR-342, and let-7g) down-regulated in healthy subjects infused with LPS (128).

##### 3.2.5.1 Anti-inflammatory activity

miR-150 targets notch receptor 1, STAT1 and NF-κB to inhibit LPS-induced apoptosis and IL-1β, IL-6 and TNF, ICAM-1, VCAM-1 and E-selectin in RAW 264.7 macrophages, THP-1 monocytic cells and endothelial cells (150, 154, 155). miR-150-5p is decreased in the heart of rats challenged with LPS. miR-150 decreases Akt2, cleaved caspase-3, Bax and apoptosis in rat heart and H9C2 cardiomyocytes (152). In a similar way, miR-150 binding to MALAT1 lncRNA inhibits the NF-κB pathway, cytokine production, ER stress and apoptosis in LPS-stimulated human umbilical endothelial cells, H9c2 cardiomyocytes, IL-1β-stimulated chondrocytes, and pulmonary arterial endothelial cells from CLP mice (150, 151, 153). miR-150-5p interacts with X-inactive specific transcript lncRNA to regulate the c-Fos axis, thioredoxin-interacting protein-mediated pyroptosis and sepsis-induced myocardial injury (157). In sepsis mice with acute kidney injury (AKI), miR-150 targets MEKK3, inhibits LPS-induced c-Jun N-terminal kinase (JNK) pathway, apoptosis and inflammation (156). miR-150^-/-^ mice show increased mortality from LPS and CLP. Rescuing miR-150 in lung endothelial cells decreases EGR2-dependent Ang2 expression, restores adherent junction reannealing and endothelial barrier function, and reduces mortality (149). miR-150 inhibits ARG1 and the expansion and immunosuppressive function of MDSCs (146) that expand during severe infections and have been associated with nosocomial infections, morbidity, and mortality in critically ill patients (17, 28, 322). miR-150-3p may have similar expression pattern and activity as miR-150-5p. miR-150-3p is one of the most downregulated exosomal miRNAs (with 146a-5p, 150-3p, 151a-3p) in heat stroke, associated with inflammatory response and coagulation cascade (323).

##### 3.2.5.2 Inflammatory activity

There is no formal demonstration of a proinflammatory activity of miR-150. Though, miR-150 is increased (and not decreased) in the serum of mice ongoing CLP-induced sepsis and in rats challenged with LPS (147, 148).

##### 3.2.5.3 Biomarker value

Many studies have reported decreased miR-150 expression in sepsis conditions. An initial miRNome study identifies 17 differentially expressed miRNAs in sepsis patients and healthy subjects (n = 17/32). miR-150 is decreased in patients. miR-150 positively correlates with diseases severity evaluated by SOFA score, and inversely correlates with cytokine levels (203). The authors propose that miR-150 could be used as a biomarker of early sepsis.

miR-150 is decreased in healthy subjects infused with endotoxin (251), and in patients with urosepsis (250), sepsis with AKI (156), and other sepsis conditions (146, 150, 204, 249). Several studies have reported negative correlations between miR-150-5p and IL-1β and TNF serum levels, renal dysfunction and T cells immunoparalysis (150, 156, 204). Accordingly, sepsis patients with fatal outcomes have reduced miR-150 levels (150, 248, 251, 252). A combination of miR-150 and SOFA score improves prognosis prediction (252). However, while patients with sepsis show lower levels of miR-150 than patients with SIRS and non-sepsis trauma patients (146, 249), no significant difference is observed between critically ill patients with and without sepsis (248). It is proposed that miR-150 may be a useful biomarker or target in the diagnosis, prognosis and treatment of sepsis. This suggestion should be tempered since miR-150 is not differentially expressed in adult sepsis patients tested for 13 miRNAs (309) and, more annoying, in unbiased studies looking at miRNome (195, 196, 198, 202).

Several reasons explain why miRNAs biomarkers are not confirmed in miRNome studies. In any case, it shows that we could increase robustness of the methodology (including cohort constitution) to accurately demonstrate miRNA differential expression in sepsis. On the other side, all studies so far reported anti-inflammatory mode of action of miR-150.

#### 3.2.6 miR-155

miR-155 is encoded on human chromosome 21. Its expression is increased by MAMPs, bacteria, viruses and parasites (267, 324–333). Captivatingly, the induction of miR-155 in macrophages is controlled by the molecular clock controller Bmal1, which in turn is repressed by miR-155. Thus, miR-155 is a regulatory component of the circadian rhythm, and of the circadian control of inflammation (267).

##### 3.2.6.1 Anti-inflammatory activity

miR-155 targets TGF-β activated kinase 1 binding protein 2 (TAB2) and negatively regulates the TLR/IL-1 signaling cascade in human DCs exposed to microbial stimuli (271). miR-155 inhibits caspase 1 and IL-1β by increasing autophagy through inhibition of TAB2. miR-155 agomir reduces lung pathology in mice with CLP (254). miR-155 inhibits IRF8-mediated antiviral response in Japanese encephalitis virus infected microglial cells (333). In *Francisella tularensis*-infected human macrophages, miR-155 downregulates MyD88 (327). The delivery of miR-155 inhibitor to mice challenged with LPS increases SOCS1, and reduces JAK and STAT3, cytokines, and kidney injury (325). In mice with CLP, a miR-155 mimic decreases JNK and β-arrestin 2 expression, reduces infiltration of macrophages and PMNs in the myocardium, and attenuates late sepsis-induced cardiac dysfunction (162). miR-155-deficient mice infected with H1N1 influenza virus and challenged 5 days later with *Staphylococcus aureus* show a robust induction of IL-17 and IL-23 and reduced bacterial burden in lungs. In a similar way, a miR-155 antagomir (*i.e.* anti-miRNAs, in the form of oligonucleotides silencing endogenous miRNAs) enhances lung bacterial clearance in mice (326). This could be relevant since post influenza bacterial pneumonia is an important cause of morbidity and mortality.

The infection of astrocytes with *Escherichia coli* induces miR-146a and miR-155 expression. In a feedback loop mechanism, miR-146a and miR-155 inhibit TLR- and epithelial growth factor receptor (EGFR)-mediated NF-κB signaling pathway and inflammation. miR-146a and miR-155 antagomirs increase brain inflammation in mice infected with *E. coli*. Thus, miR-155 acts coordinately with miR-146a to safeguard the central nervous system from neuroinflammatory damages (334).

##### 3.2.6.2 Inflammatory activity

Pioneer studies published in late 2000’s linked miR-155 with inflammation and innate immunity. miR-155 has been identified as a target induced by inflammatory mediators in macrophages (324). Subsequently, miR-155 is shown to repress SOCS1 and Src homology 2 domain containing inositol polyphosphate 5-phosphatase 1 to increase LPS-induced cytokine production by mouse macrophages (269, 270).

miR-155 transgenic mice produce more TNF in response to LPS and are more sensitive to endotoxemia (335). miR-155 deficient mice have a reduced capacity to clear *Streptococcus pneumoniae* colonization from the nasopharynx, which is associated with impaired recruitment of macrophages and induction of protective T helper (Th) 17 immune responses (328). PMNs from miR-155-deficient septic mice express less NETs. miR-155 deficiency is associated with reduced accumulation of PMNs, NETs, edema and lung damage in mice with CLP (158). miR-155 is increased in endothelial cells from endotoxemic mice, and in the serum and bronchoalveolar lavage fluid (BALF) from septic patients with ARDS. miR-155 promotes vascular permeability and capillary leakage (159). miR-155 deficiency reduces endothelial activation and leukocyte adhesion and infiltration into the myocardium, myocardial edema and dysfunction, vasoplegia, and mortality in mice with endotoxemia or CLP. miR-155 targets CD47 and angiotensin type 1 receptor to promote nitric oxide (NO)-mediated vasorelaxation and vasoplegia (161). Injection of a miR-155 inhibitor reduces inflammation and intestinal barrier dysfunction in mice with CLP (160).

##### 3.2.6.3 Biomarker value

The measure of 13 miRNAs in the plasma of 32 healthy controls and 62 patients with sepsis shows that 11 miRNAs including miR-155 are increased in patients. miR-155 levels are not associated with severity or outcome (309). A miRNome identifies 11 differentially expressed miRNAs in sepsis patients compared to healthy controls (n = 60/30), but only miR-155 is confirmed by PCR. miR-155 is elevated in patients, and positively correlates with SOFA score. miR-155 shows a good prediction value of 28-day survival (AUC = 0.763). Interestingly. miR-155 levels are proportional to the percentage of CD39^+^ regulatory T cells (201). Another study reports that miR-155 is increased in septic patients and is a valuable predictor of mortality (241). In a study analyzing 10 healthy controls and 10 sepsis patients with ARDS, miR-155 levels are elevated in BALF samples from sepsis patients (254). In a cohort of 156 sepsis patients of whom 41 with ALI and 32 with ARDS, miR-155 levels are higher in patients with ALI or ARDS, positively correlate with IL-1β and TNF, and negatively correlate with PaO2/FiO_2_ ratio. miR-155 AUC for diagnosing sepsis with ALI/ARDS is 0.87 (253). A study comparing 218 critically ill patients (135 with sepsis) with 76 healthy controls shows that, in critically ill patients ≤ 65 years, high miR-155 levels are associated with increased survival. This is not the case in patients older than 65 years (336). Finally, miR-155 is similarly expressed in peripheral blood from newborns with or without sepsis (237).

To summarize, there are strong arguments in favor of anti-inflammatory and proinflammatory activities of miR-155. miR-155 is usually increased in adults with sepsis, and associated with worse outcome. This is not observed in elderly and newborns, suggesting that miRNA-based biomarkers should be interpreted according to patient’s age.

#### 3.2.7 miR-223

miR-233 is encoded on chromosome X in mammals, and is highly conserved among species. miR-223 regulates hematopoiesis and triggers granulopoiesis and macrophage differentiation (337–340). miR-223 targets NLRP3, IGF1R, HSP90, C/EBPα, C/EBPβ, E2F1, forkhead box protein O1, NF-κB p65, nuclear factor I A, PBX/knotted 1 homeobox 1, STAT3 and STAT5, which accounts for a broad range of biological effects (338).

##### 3.2.7.1 Anti-inflammatory activity

miR-223 is predominantly expressed in myeloid cells and drives anti-inflammatory functions. miR-223 is involved in macrophage polarization and activation, and negatively regulates neutrophil functions. miR-223 inhibits NF-κB p65 phosphorylation and IL-1β, IL-6, TNF and IL-12p40 expression in U-937 human monocytic cells stimulated with LPS and IFNγ (341). NLRP3 is a sensor of the classical inflammasome involved in gasdermin-D processing, pyroptosis and secretion of IL-1β and IL-18 (342). miR-223 suppresses NLRP3 expression and IL-1β production in mouse macrophages and PMNs (343). Stimulation of macrophages with LPS, CpG DNA or poly(I:C) decreases miR-233 expression, which results in increased STAT3, NF-κB and MAPK signaling and production of IL-1β, IL-6 and TNF (344, 345). In the same line, PMN-derived miR-223 inhibits NLRP3 and IL-1β expression, and reduces pathogenesis in mice with DAMPs-induced ALI (346). miRNA-223 is upregulated in blood and lung parenchyma during experimental and human tuberculosis (347), and in monocytes from patients with tuberculosis (341). In a mouse model, miR-223 restricts the expression of CCL3, CXCL2 and IL-6 and the recruitment of PMNs into the lungs. miR-223 knockdown sensitizes mice to *Mycobacterium tuberculosis* lung infection through exacerbated PMN-dependent lethal inflammation (347). miR-223 promotes MMP-1 and MMP-9 activity in macrophages. *M. tuberculosis* infection increases the expression of miR-223, MMP-1 and MMP-9 in lungs. In doing so, it favors bacteria dissemination. On the contrary, miR-223 impedes BMAL1, which influences the expression of the circadian clock genes CLOCK, PER1 and PER2. Thus, *Mycobacterium tuberculosis* interferes with circadian rhythm *via* a miR-223/BMAL1 axis to subvert host defenses (348). Mechanical ventilation and *Staphylococcus aureus-*induced ALI is increased in miR-223 deficient mice. Pulmonary delivery of miR-223 using nanoparticles inhibits ALI. Interestingly, the transfer of miR-223 from PMNs to alveolar epithelial cells may be involved in attenuating lung inflammation (349).

##### 3.2.7.2 Inflammatory activity

miR-223 increases in lungs of mice exposed to cigarette smoke and LPS and human in pulmonary cells and monocytes exposed to inflammatory cytokines. miR-223 targets histone deacetylase 2 (HDAC2), resulting in increased expression of fractalkine. miR-223 negatively correlates with HDAC2 expression in lungs from chronic obstructive pulmonary disease (COPD) patients (181). High miR-223 levels might contribute to stimulate the NF-κB pathway, and decrease corticosteroid response and disease severity in asthma and COPD (350).

##### 3.2.7.3 Biomarker value

Studies evaluating miR-223 as a sepsis biomarker have generated contradictory results. When compared to healthy controls, miR-223 serum levels are either reduced (197, 237, 242), increased (182, 199, 204, 207) or not affected (259, 309) in patients. Observations using severity as a variable appear more consistent since miR-223 levels are lower in sepsis patients than in SIRS patients (242), and in patients with sepsis-induced cardiomyopathy than in healthy controls (238). Yet, miR-223 levels are either lower (182, 195) or higher (260) in sepsis non-survivors than in sepsis survivors (351). Finally, miRNome studies have not pointed to miR-223 as a differentially expressed miRNA in sepsis (195, 196, 198, 202).

Overall, miR-223 is considered anti-inflammatory, albeit it might drive inflammatory effects by targeting HDAC2 in specific conditions. Clinical studies yielded heterogeneous results when assessing the potential of miR-223 as a biomarker of sepsis. However, a meta-analysis of 22 records, including 2210 sepsis, 426 SIRS, and 1076 healthy controls suggested that miR-223 could be used as an indicator for sepsis (351). It should be stressed however that miR-223 values were available in a subset of 6/22 studies.

#### 3.2.8 Other miRNAs

Finally, we will describe few studies analyzing miRNAs in an unsupervised manner or in the context of specific clinical questions. A miRNome analysis in critically ill patients with intra-abdominal sepsis or non-infective SIRS and healthy controls (n = 29/44/16) has detected 116 blood miRNAs increased in SIRS patients. miRNAs are more abundant in non-infectious SIRS than in sepsis patients. The top five differentially expressed miRNAs, miR-23a-5p, miR-26a-5p, miR-30a-5p, miR-30d-5p and miR-192-5p, discriminate severe sepsis from severe SIRS (AUC = 0.74-0.92). miRNA levels inversely correlate with IL-1, IL-6, IL-8, CRP and pancreatic stone protein (PSP), but not SOFA score. Hence, sepsis and non-infective SIRS are characterized by distinct changes in blood miRNAs, which may be used for diagnostic approaches in critically ill patients (196). However, except miR-23a, none of the short listed miRNAs are considered as sepsis biomarkers in previous studies (89, 249).

A recent study evaluated blood changes of miR-15a-5p, miR-155-5p, miR-192-5p, miR-423-5p in 46 sepsis patients treated with gentamicin, vancomycin (*i.e.* nephrotoxic antibiotics) or non-nephrotoxic antibiotics (n = 20/7/19). Small changes of miRNAs are observed in the different groups. miR-15a-5p at day 7 of gentamicin treatment provides good discrimination between AKI and non-AKI. miR-155-5p and miR-192-5p positively correlate with creatinine and neutrophil gelatinase-associated lipokalin in patients receiving vancomycin (210). These data suggest that miRNAs expression might be modulated by antimicrobials, and may serve as diagnostic markers in sepsis patients receiving nephrotoxic antibiotics.

The expression of miR-146-3p, miR-147b, miR-155 and miR-223 (associated with inflammation, see **3.2**) was assessed in the plasma of patients with bacterial sepsis or dengue hemorrhagic fever and healthy controls (n = 130/69/82). miRNAs are increased in patients with sepsis when compared to patients with hemorrhagic fever or to healthy controls. miR-147b, alone or in combination with PCT, discriminates septic shock (AUC ≥ 0.8). Thus, miR-147b may be a biomarker to support clinical diagnosis of severe sepsis (247).

Necrotizing enterocolitis (NEC) is the most common and severe gastrointestinal pathology in preterm infants. A microarray-based screening has identified 230 upregulated miRNAs and 16 downregulated miRNAs in NEC when compared to sepsis and non-NEC/non-sepsis groups. Targeted analyses in a large cohort shows that miR-1290 can efficiently differentiate NEC from neonatal sepsis and neonatal inflammatory conditions such as bronchopulmonary dysplasia (200). Plasmatic miR-1290 expression may help differentiating NEC from neonatal sepsis.

## 4 Conclusions

Over the past decade, miRNAs have been the focus of intense research in the field of critical illness and sepsis. Our understanding of the modes of action and impact of miRNAs on host inflammatory and antimicrobial defenses has increased dramatically. However, this has not yet improved clinical management. Possibly, intervention strategies with miRNA mimics or miRNA antagomirs could rebalance the dysregulated host response during sepsis (**Figure 1**). Unfortunately, no miRNA-based clinical trials have been registered for sepsis so far.

The data summarized in **Table 1** and **Figure 3** illustrate the complexity and wide range of action of miRNAs in inflammatory and infectious conditions. Some miRNAs have been ascribed both anti-inflammatory and proinflammatory activities. Many reasons may account for diverse observations, including differences between *in vitro*, *ex vivo* and *in vivo* settings, sterile and infectious models, organs and cell types examined, and kinetics. In *in vivo* sepsis models, a mediator may be beneficial or harmful depending on disease condition. For example, inhibition of macrophage migration inhibitory factor (a pleiotropic cytokine and central regulator of innate immune responses (352, 353) increased susceptibility to infection but protected from lethal sepsis (354–357). Similarly, blocking TLR4 at the onset of infection induced mortality from otherwise non-lethal peritonitis, while therapeutic administration of anti-TLR4 antibodies protected mice from lethal Gram-negative bacterial sepsis (299).

Using miRNA as biomarker in sepsis holds more short-term potential than therapeutic opportunities. Many studies reported that miRNAs: 1) discriminate healthy donors from sepsis patients, 2) distinguish sepsis from non-infectious clinically-related diseases, 3) predict severity and/or the mortality, and 4) correlate with clinical parameters or cytokines. However, conflicting observations currently make translation to clinics challenging. So, how to use more efficiently miRNAs as biomarkers?

There is a crucial need for improvement and standardization of clinical studies in order to generate comprehensive views of miRNome during sepsis. We advocate for more stringent methodologies, in terms of both study design, clinical data collection, and miRNA investigation strategies. Importantly, small cohorts tends to exacerbate individual variations, whereas targeted techniques (e.g. RT-qPCR) fail to generate a global landscape of the miRNA fluctuations. Even if constraining, derivation and validation cohorts should be envisaged to corroborate and improve robustness of observations. A key objective would be to run unbiased miRNome analyses in large cohorts of well-defined critically-ill patients with or without sepsis.

Sepsis is a heterogeneous syndrome. Mediators detrimental during the overwhelming phase of sepsis might be beneficial during the later immunosuppressive phase, and miRNAs should not deviate from this principle (**Figure 1**). In fact, new types of clinical trials using a precision-medicine approach have been launched to adjust treatment (immunosuppressive or immuno-stimulant) given patients’ inflammatory status (see https://www.immunosep.eu/ as an example). We believe that studies should take into consideration the causative agent, the site of infection, medications and the inflammatory status to stratify patients. Bearing in mind disease progression, the timing of sampling should be recorded. Ideally, blood samples should be collected at hospital admission (ED, medical/surgical ICUs), and continued over time to have a longitudinal view of the expression miRNAs. For translational perspectives, it would be easier be detected miRNAs in serum or blood than in PBMCs or isolated vesicles.

Finally, miRNA expression levels are prone to be affected by individual parameters (age, sex, genetic, comorbidities…). Therefore, combination scores (including one or several miRNAs, demographic and/or clinical data) should also be considered. Along these lines, whole blood or single cell transcriptomic identified rather simple gene expression signatures to distinguish sterile inflammation from sepsis, sepsis from infection, viral infections from fungal and bacterial infections, peritonitis, and sepsis caused by community-acquired pneumonia (358–363). Ideally, polymorphisms affecting pri-, pre- and mature miRNA sequences or affecting the target gene sequence should be investigated as well (364).

Based on our current knowledge, clinical use of miRNA targeting in sepsis cannot be realistically envisaged. miRNAs might be used as biomarkers. However, further studies will be required to obtain robust results, in order to safely recommend the use of miRNAs as biomarkers of sepsis. This is a certainly an ambitious, but promising goal.

## Author contributions

TR conceived the manuscript. TR and NA wrote the manuscript. NA, CG, CT, ITS and TR revised the manuscript. All authors contributed to the article and approved the submitted version.

## Funding

TR is supported by the Swiss National Science Foundation (SNSF, grant number 310030_207418), by the Horizon 2020 Marie Skłodowska-Curie Action: Innovative Training Network (MSCA-ESA-ITN, grant number 676129) and Horizon 2020 ImmunoSep (grant number 847422), by the Fondation Carigest/Promex Stiftung für die Forschung (Geneva, Switzerland) and the Fondation de Recherche en Biochimie (Epalinges, Switzerland). NA received a scholarship from the Porphyrogenis Foundation (Lausanne, Switzerland). CT and IS received a scholarship from the Société Académique Vaudoise (Lausanne, Switzerland).

## Acknowledgments

We apologize for those studies that were not mentioned in this review.

## Conflict of interest

The authors declare that the research was conducted in the absence of any commercial or financial relationships that could be construed as a potential conflict of interest.

## Publisher’s note

All claims expressed in this article are solely those of the authors and do not necessarily represent those of their affiliated organizations, or those of the publisher, the editors and the reviewers. Any product that may be evaluated in this article, or claim that may be made by its manufacturer, is not guaranteed or endorsed by the publisher.

## Abbreviations

AKI, acute kidney injury; ALI, acute lung injury; APACHE, acute physiology and chronic health evaluation; ARDS, acute respiratory distress syndrome; CCL, C‐C motif chemokine ligand; CXCL, C‐X‐C motif chemokine ligand; DAMP, damage or danger-associated molecular pattern; DC, dendritic cell; HMGB1, high mobility group box-1; HSP, heat shock protein; ICU, intensive care unit; IFN, interferon; IL, interleukin; IRF, IFN response factor; IRAK, IL-1 receptor-associated kinase; lncRNA, long non-coding RNA; LPS, lipopolysaccharide; MAMP, microbial-associated molecular pattern; MAPK, mitogen-activated protein kinase; miRNA, microRNA; MyD88, myeloid differentiation primary response 88; NF-κB, nuclear factor-κB; PCT, procalcitonin; PRR, pattern-recognition receptor; SIRS, systemic inflammatory response syndrome; SOCS, suppressor of cytokine signaling; SOFA, sequential organ failure assessment; STAT, signal transducer and activator of transcription; TLR, Toll-like receptor; TNF, tumor necrosis factor.
